# Supplementary material for: Mixed-methods analysis of select issues reported in the 2016 World Health Organization verbal autopsy questionnaire
Source: PLoS One. 2022 Oct 7;17(10):e0274304. doi: 10.1371/journal.pone.0274304 (PMC9543875; doi:10.1371/journal.pone.0274304)
Supplement: S1 File — (DOCX) [file pone.0274304.s001.docx]

**Mixed-Methods Analysis of Select Issues Reported**

**in the 2016 WHO VA Questionnaire:**

**Supplement A**

**TABLE OF CONTENTS**

[BACKGROUND 3](#_Toc60234779)

[METHODS 3](#_Toc60234780)

[RESULTS 6](#_Toc60234781)

[1. TOBACCO USE 7](#_Toc60234782)

[2. SWALLOWING 9](#_Toc60234783)

[3. SORES AND ULCERS 11](#_Toc60234784)

[4. SWELLING, LUMP, ULCERS, PITS IN THE BREAST 13](#_Toc60234785)

[5. OTHER FEMALE HEALTH RELATED QUESTIONS 16](#_Toc60234786)

[6. DIAGNOSIS BY A HEALTH PROFESSIONAL VS SYMPTOM REPORT 20](#_Toc60234787)

[7. VACCINATIONS 32](#_Toc60234788)

[8. INJURY QUESTIONS 34](#_Toc60234789)

[9. URINE 36](#_Toc60234790)

[10. ABDOMINAL PROBLEM 38](#_Toc60234791)

[11. LUMPS 41](#_Toc60234792)

[12. VOMITING 44](#_Toc60234793)

[13. VIOLENCE 46](#_Toc60234794)

[14. BABY SIZE 4](#_Toc60234795)9

# BACKGROUND

Verbal autopsy (VA) is a method for estimating population-level cause of death patterns for mortality surveillance purposes; information is obtained from the caretaker of a deceased person, whereby trained interviewers visit the relatives of the deceased to conduct VA interviews using electronic data capture instruments or paper questionnaires. Information obtained during VA interviews include the circumstances, signs, and symptoms during the terminal stage to find out the likely cause of death, health care seeking in the period leading to death, and history of events leading to death as narrated by the respondent. Cause of death determination from VA can be done using physicians review or using automated computer algorithms.

In 2007, WHO introduced the first international technical standards and guidelines for VA; the current version is the 2016 WHO VA instrument, which is now used by more than 20 countries. We created a public facing repository: <https://github.com/SwissTPH/WHO_VA_2016>. This is connected to a WHO email address that users can email to report issues. The repository was created and is maintained by Swiss TPH, a Collaborating Center partner of the WHO VA Reference Group. The public version is targeted for VA users to report and track responses on reported issues. Use of GitHub started in mid 2017. Issues reported through late 2019 were incorporated in the analysis. Issues reported with its use have been compiled and a major revision of the instrument is planned based on users’ feedback and evidence from the field. Among the chief feedback received is the need to shorten the questionnaire in order to facilitate routine application.

Accordingly, the WHO VA Reference Group (VARG) has undertaken the development of an international standard VA questionnaire that is the best possible for use in routine mortality surveillance. Such an instrument needs to: 1) be concise, clear and efficient, 2) perform well in the field, and 3) be compatible with automated algorithms for assigning probable cause of death.

The objective of this activity is to:

Apply a mixed methods analysis approach to known issues with the 2016 WHO VA instrument using available evidence on the performance of the existing questionnaire to

1. Highlight areas that may be contributing to the underperformance of an item and

2. Identify potential question series or items to target for revision

# METHODS

This activity included mixed methods analysis of secondary data collected using the 2016 WHO VA questionnaire together with cognitive interviewing results.

*Secondary Data*

For quantitative analysis, de-identified VA questionnaire data was provided from teams representing five countries, including government or research teams that are known to have compiled at least 1,000 VAs using the 2016 WHO VA questionnaire (https://www.who.int/healthinfo/statistics/verbalautopsystandards/en), along with the Child Health and Mortality Prevention Surveillance (CHAMPS) and Countrywide Mortality Surveillance for Action (COMSA) Initiatives. All data were collected electronically. Submitted data from the VA questionnaires were compiled into two datasets for this analysis -- the primary dataset, which includes all data submitted, and the reference dataset, which includes VA data and PCVA-assigned causes of death. More details on these datasets follow.

The primary dataset consists of aggregated VA results using the 2016 WHO Verbal Autopsy questionnaire submitted by the following countries and sources: Zambia (VAs conducted by the Department of National Registration, Passports, and Citizenship for community deaths “brought in dead” to two mortuaries in Lusaka, Zambia); South Africa (as described above), Kenya (VAs from the KEMRI/CDC Health and Demographic Surveillance Site in Western Kenya), CHAMPS and COMSA. The CHAMPS Initiative focuses on mortality surveillance in children under 5 in Sub-Saharan Africa and Southeast Asia. Participating countries include Bangladesh, Mali, Mozambique, Ethiopia, Kenya and South Africa. COMSA is a surveillance program in Mozambique that produces and makes publicly available continuous annual data on mortality and cause of death at national and subnational levels within the country. The primary dataset includes 19,150 verbal autopsies: 13,736 adults, 2,916 children and 2,498 neonates; 10,280 are males and 8,870 are females.

The reference dataset contains 5,389 verbal autopsies: 102 neonates, 187 children and 5,100 adults; 2,579 are female and 2,810 are male. The reference dataset will be used to explore cause of death data.

The qualitative information used in this activity was secondary analysis of results from cognitive interviews conducted in Zambia and Morocco in 2019, with support from the Collaborating Center for Questionnaire Design and Evaluation Research (CCQDER) at CDC’s National Center for Health Statistics (NCHS). Cognitive interviewing is a qualitative method whose purpose is to evaluate survey questionnaires and determine which constructs the questionnaires’ items capture. The primary benefit of cognitive interviewing over non-qualitative evaluation methods is that it provides rich, contextual data into how respondents interpret questions, apply their lived experiences to their responses, and formulate responses to survey items based on those interpretations and experiences. Thus, cognitive interviewing data allows researchers and survey designers to understand whether or not a question is capturing the specific social constructs they originally wanted and gives insight into what design changes are needed to advance the survey’s overall goal.

Information was collected from cognitive interviews with living respondents about deceased individuals; NCHS staff trained the local interviewers to conduct the cognitive interviews and compiled and analyzed the cognitive interviewing results. In the 2019 evaluation of the VA questionnaires, a purposive sample of 149 respondents across two sites—Lusaka, Zambia and Rabat, Morocco—was recruited to participate in cognitive interviews. An effort was made to create a sample with a range of decedent ages, so that all three questionnaires could be evaluated fully. Unfortunately, given the specifics of how respondents were recruited in Morocco, proportionally fewer respondents from that country received either the child or neonate questionnaire as compared to the Zambian sample. As a result, the final sample for this project is slightly skewed towards respondents who received the adult questionnaire, as shown in Table 1:

**Table 1: Cognitive Interviewing Sample by Country and Questionnaire**

|  | **Adult** | **Child** | **Neonate** | ***Total*** |
| --- | --- | --- | --- | --- |
| **Morocco** | 45 | 20 | 19 | 84 |
| **Zambia** | 23 | 23 | 19 | 65 |
| ***Total*** | 68 | 43 | 38 | 149 |

Respondents in Zambia were sampled at a morgue at a city hospital. With the support of the Zambian government, VAs are conducted for all brought-in-dead (i.e. decedents who did not die in a hospital) in Lusaka. Cognitive interviewers recruited respondents from the pool of verbal autopsy respondents, and the cognitive interviews were conducted directly following the VA. In Morocco, VAs are conducted for non-hospital deaths at the Ministry of the Interior’s Vital Registration Office when family members come to register a death and obtain a burial certificate. Just as in Zambia, cognitive interviewers recruited respondents from this pool of VA respondents and conducted the cognitive interview directly following the VA. Incentives were not provided to respondents in either location.

Cognitive interviewers entered their notes into CDC’s Q-Notes software[1], which is a qualitative analysis program designed specifically for the storage and analysis of data from cognitive interviews. Following a week-long training course conducted by NCHS researchers, the local cognitive interviewers conducted the interviews over a period of four months (for Zambia) and one year (for Morocco). NCHS researchers were able to monitor the data collection and data quality via Q-Notes and communicated with the field teams when necessary to provide direction and assistance.

*Analysis*

Analysis focused on 14 issues of clarity or redundancy that have been reported by users of the 2016 WHO VA questionnaire through a GitHub repository and flagged for review as part of the next major instrument revision (<https://github.com/SwissTPH/WHO_VA_2016>). To identify areas of redundancy, inconsistency or overlap among these issues, frequency distributions and cross tabulations of quantitative interview data were run to compare response patterns in related questions within a series. Impact of respondent characteristics on response patterns were further evaluated by calculating prevalence ratios, 95% confidence intervals and *p-*values*.*

The qualitative cognitive interviewing results were reviewed for additional understanding of 1) known issues with the questionnaire that have been reported by users and 2) issues that emerge from the quantitative analysis described above. The cognitive interviewing report was also reviewed to identify any new issues that were further explored in the quantitative data; Of note, not all issues identified through the repository were addressed in the cognitive interviews, therefore cognitive testing input was applied where available

*Presentation of Results*

Each of the 14 issues are presented in a separate section below. Each section begins with a brief description of the issue and a shaded table that shows the questions of concern and the item sequence; first order questions are shaded in dark green, with graded lighter shading of second and third order questions. First order questions are asked of all respondents. Second and third order questions are only asked dependent on the response to the first (or second) order question. For example, a “Yes” response to a first-order question might then trigger second- and third-order questions exploring this “Yes” response in further detail; whereas those who answered “No” to this first order question, would not be asked the second-order questions.

A results section is provided for each issue presenting the qualitative and quantitative results. Quantitative results include frequency tables, cross tabulations and prevalence ratios as described above. Qualitative results of cognitive interviews are presented where available.

The results of this analysis are meant to provide evidence to inform decisions around the revision of the standard verbal autopsy instrument. This document is not intended to provide interpretations, recommendations or conclusions from these results.

The following documents were referenced in compiling the evidence about the question patterns in this review:

- 2016 WHO VA electronic questionnaire file: WHOVA2016_v1_5_2_XLS_form_for_ODK (available from <https://www.who.int/healthinfo/statistics/verbalautopsystandards/en/>)
- VA Field Interviewer Manual

(available from <https://www.who.int/healthinfo/statistics/verbalautopsystandards/en/>)

##

##

## 1. TOBACCO USE

**ISSUE**

The series contains 5 questions exploring the use of tobacco: 2 required, first-order question and 3 second or third order questions. Can the question series shown in Table 1.1 be shortened?

**Table 1.1 Question series, asked for adults**

| **Question** | **Comments** |
| --- | --- |
| Did (s)he use tobacco? | Required |
| Did (s)he smoke tobacco? | Required |
| What kind of tobacco did (s)he use? | If “Yes” to first order question |
| How many cigarettes did (s)he smoke daily? | If “Yes” to first order question |
| How many times did (s)he use tobacco products each day? | Asked if selected ‘pipe’, ‘chewing tobacco’, 'local form of tobacco', or 'other' in response to *“What kind of tobacco did (s)he use?”* |

**QUANTITATIVE ANALYSIS**

**A.**

The questions, *“Did (s)he use Tobacco?”* and *“Did (s)he smoke tobacco?”* are similar, though slightly different questions. Table 1.2 shows the response patterns for these two questions.

**Table 1.2 Agreement between “Did (s)he use Tobacco?” and “Did (s)he smoke tobacco?”**

| **Did (s)he smoke tobacco?** | | | | |  |
| --- | --- | --- | --- | --- | --- |
| **Did (s)he use tobacco?** | **Yes**  **n**  **(Row% / Column%)** | **No**  **n**  **(Row% / Column%)** | **Don’t know**  **n**  **(Row% / Column%)** | **Refused** | **Total** |
| **Yes** | 3,097  (95% / 95%) | 43  (1.3% / 0.5%) | 111  (3% / 45%) | 0 | 3,251 (24%) |
| **No** | 151  (1.6% / 4.6%) | 9,525  (97% / 99%) | 46  (0.5%/19%) | 0 | 9,722 (74%) |
| **Don’t know** | 3  (2.4%/ 0.09%) | 35  (28% / 0.4%) | 87  (70%/36%) | 0 | 125 (1%) |
| **Refused** | 0 | 0 | 0 | 3 (100%) | 3 (<1%) |
| **Total** | 3,251  (25%) | 9,603  (73%) | 244  (2%) | 3  (<1%) | 13,101 |

**B.**

*“Did (s)he use Tobacco?”* and *“Did (s)he smoke tobacco?”* are similar. Is the follow up question, *“What kind of tobacco did (s)he use?*” able to capture tobacco smokers if the question, *“Did (s)he smoke tobacco?”* were eliminated? Table 1.3 shows the response patterns for the two questions.

**Table 1.3 Agreement between “Did (s)he use tobacco?” and “What kind of tobacco did (s)he use?”**

| **What kind of tobacco did (s)he use?** | | | | | |  |
| --- | --- | --- | --- | --- | --- | --- |
| **Did (s)he use tobacco** | **Chewing Tobacco**  **#**  **(row% / col%)** | **Cigarettes** | **Local Form of Tobacco** | **Other** | **Pipe** | **Total** |
| **Yes** | 19  (0.6% / 87%) | 2,729  (89% / 96%) | 248  (8% / 95%) | 10  (0.3% / 77%) | 69  (2% / 91%) | 3,075 (95%) |
| **No** | 0 | 124  (86% / 5%) | 11  (8% / 4%) | 3  (2% / 23%) | 7  (5% / 10%) | 145  (5%) |
| **Don’t Know** | 0 | 2  (67% / 0.1%) | 1 | 0 | 0 | 3 |
| **Total** | 19  (0.6%) | 2,855  (89%) | 260  (8%) | 13  (0.4%) | 76  (2%) | 3,223 |

**C.**

*“How many cigarettes did (s)he smoke daily?”* as an open ended question that requires a specific response that may be difficult for respondents to provide as a proxy to the deceased*.*

**Table 1.4 Response patterns for “How many cigarettes did (s)he smoke daily?”**

|  | **Number** | **Percent** |
| --- | --- | --- |
| **<10** | 543 | 23% |
| **≥ 10** | 344 | 14% |
| **Unknown** | 1,516 | 63% |
| **Total** | 2,403 | 100% |

**QUALITATIVE OBSERVATIONS**

- In the cognitive testing sample, all who used tobacco *(“Did (s)he use Tobacco?”),* smoked it *(“Did (s)he smoke tobacco?”).* There was no one who used tobacco in some other way that was not smoking. There was one mismatch, or response error, between the questions, but the reason is not known. All that is known from the data is that the deceased quit smoking cigarettes years ago (“No” to “*Did (s)he use Tobacco?*”, “Yes” to *“Did (s)he smoke tobacco?*”). Answers also varied for those who had quit smoking. Some answered “Yes” to the questions on using and smoking tobacco, and others answered no.
- “*What kind of tobacco did s/he use?”* also did not pick-up any new information. All those who used tobacco smoked cigarettes.
- The similarity of questions, *“Did (s)he use tobacco?”, “Did (s)he smoke tobacco?*” and “*What kind of tobacco did (s)he use?”* was confusing to respondents: “He used to smoke cigarettes as I said, the respondent answered.” In general, when respondents are confused about the similarity among questions, they attach meaning to this phenomenon. Possible meanings include “I must have misunderstood one of the questions” and “they are trying to trick me.” This can lead to response errors.
- “*How many cigarettes did (s)he smoke daily?”* and *“How many times did (s)he use tobacco products each day?”* were also perceived as very similar to respondents. Many people did not know how many cigarettes the deceased smoked. *“How many times did (s)he use tobacco products each day?”* did not pick up any new information; either respondents just repeated that they did not know, or they gave the same answer as they did for *“How many cigarettes did (s)he smoke daily?”.* The similarity of these questions could also lead to confusion and response errors.
- Better response rates are typically seen when a specific option in the question is provided rather than an open ended (e.g., how many) question.

## 2. SWALLOWING

**ISSUE**

What is the consistency in responses to questions *“Did (s)he have difficulty swallowing?”* and *“Did (s)he have pain with swallowing?”* ? There may be confusion between the constructs of “difficulty” and “pain.” Are respondents able to differentiate these constructs? Can/should one question be eliminated? If both constructs are critical, do we need to better distinguish these questions, or can we consider subsetting one question within the other? Table 2.1 shows the question series; both questions are required to be asked of respondents; there is no dependence on the responses to one for the other to be asked. Additional questions are asked if the respondent answered “Yes” to difficulty swallowing.

**Table 2.1 Question series, asked for adults and children**

| **Question** | **Comments** |
| --- | --- |
| Did (s)he have difficulty swallowing? | Required |
| For how long before death did (s)he have difficulty swallowing? | If “Yes” to the first order question  units: days, months |
| [Enter how long before death (s)he had difficulty swallowing in days] | If “days” to second order question |
| [Enter how long before death (s)he had difficulty swallowing in months] | If “months” to second order question |
| Was the difficulty with swallowing with solids, liquids, or both? | If “Yes” to the first order question |
| Did (s)he have pain with swallowing? | Required |

**QUANTITATIVE ANALYSIS**

**A.**

Table 2.2 shows the agreement between *Did (s)he have pain with swallowing? and “Did (s)he have difficulty swallowing*?”

**Table 2.2 Agreement between *“Did (s)he have difficulty swallowing*?”** **and** **“*Did (s)he have pain with swallowing?”***

| **Pain** | | | |  |
| --- | --- | --- | --- | --- |
| Difficulty | **Yes**  # (Row% / Col%) | **No**  # (Row% / Col%) | **Don’t know** | **Total** |
| **Yes** | 2,052  (62% / 91%) | 891  (27% / 7%) | 346  (11% / 60%) | 3,289 (22%) |
| **No** | 198  (2% / 8%) | 11,309  (97% / 92%) | 87  (1% / 15%) | 11,594 (77%) |
| **Don’t Know** | 2  (1% / 1%) | 46  (24% / 1%) | 146  (75% / 25%) | 194 (1%) |
| **Total** | 2,252 (15%) | 12,246 (81%) | 579 (4%) | 15,077 |

**QUALITATIVE OBSERVATIONS**

- The data from cognitive interviews suggest that there is a difference in the two constructs of difficulty and pain, but they are related. Pain was a subcategory within the broader theme of “difficulties.”

1) everyone who responded “No” to difficulty swallowing also responded “No” to pain; and

2) some respondents answered “Yes” to difficulty but “No” to pain.

From cognitive interviews, other difficulties included:

- 1. Paralysis or inability to swallow
  2. Mass/lump in throat
  3. Laying down/bed ridden
  4. Sores in mouth
  5. Unknown [especially if just stopped eating and swallowing within last few days of death]
- The data suggest that it may be possible to screen out the question about pain with a “no” response to difficulties. Those who respond “Yes” to difficulties, however, should follow up with the question on pain.

## 3. SORES AND ULCERS

**ISSUE**

This question series asks multiple questions about similar though different constructs. Are the constructs clearly understood? Can the question series be shortened?

What is the consistency of responses to *“Did (s)he have sores or ulcers anywhere on the body?” and* “*Did (s)he have sores*?”, and *“Did (s)he have an ulcer (pit) on the foot?”* ? All questions are currently always asked.

**Table 3.1 Question series**

| **Question** | **Comments** |
| --- | --- |
| Did (s)he have sores or ulcers anywhere on the body? | Required for adults and children |
| Did (s)he have sores? | Required for adults |
| Did the sores have clear fluid or pus? | If ”Yes” to first order question |
| Did (s)he have an ulcer (pit) on the foot? | Required for adults and children |
| Did the ulcer on the foot ooze pus? | If “Yes” to first order question |
| How long did the ulcer on the foot ooze pus? | If “Yes” to first order question |
| [Enter how long the ulcer on the foot oozed pus in days]: | If “days” to second order question |
| [Enter how long the ulcer on the foot oozed pus in months]: | If “months” to second order question |

**QUANTITATIVE ANALYSIS**

**A.**

Table 3.2 shows the response patterns for “*Did (s)he have sores or ulcers anywhere on the body?”* and *“Did (s)he have sores?”.*

**Table 3.2 Agreement between “*Did (s)he have sores or ulcers anywhere on the body?”* and “*Did (s)he have sores?”* (adults only)**

|  | Sores | | |  |  |
| --- | --- | --- | --- | --- | --- |
| **Sores or ulcers anywhere on the body** | **Yes**  **#**  **(Row% / Col%)** | **No** | **Don’t Know** | **Refused** | **Total** |
| **Yes** | 786  (80% / 81%) | 190  (19% / 2%) | 3  (0.3% / 2%) | 0 | 979 (9%) |
| **No** | 184  (2.0% / 19%) | 9,644  (97% / 97%) | 78  (1% / 56%) | 0 | 9,906 (90%) |
| **Don’t Know** | 4  (4% / 0.4%) | 47  (43% / 1%) | 58  (53% / 42%) | 0 | 109 (1%) |
| **Refused** | 0 | 0 | 1  (33%, <1%) | 2  (67%, 100%) | 3 (<1%) |
| **Total** | 974 (9%) | 9,881 (90%) | 140 (1%) | 2 (<1%) | 10,997 |

Table 3.3 shows the response patterns for “*Did (s)he have sores or ulcers anywhere on the body?”* and *“Did (s)he have an ulcer (pit) on the foot?”.*

**Table 3.3 Agreement between “*Did (s)he have sores or ulcers anywhere on the body?”* and *“Did (s)he have an ulcer (pit) on the foot?”* (adults and children)**

| Pit/ulcer on the foot | | | |  |
| --- | --- | --- | --- | --- |
| **Sores or ulcers anywhere on the body** | **Yes**  **#**  **(Row% / Col%)** | **No** | **Don’t know** | **Total** |
| **Yes** | 399  (24% / 73%) | 1,237  (75% / 8%) | 11  (1% / 10%) | 1,647 (10%) |
| **No** | 144  (1% / 26%) | 14,517  (99% / 91%) | 49  (0.3% / 42%) | 14,710 (88%) |
| **Don’t Know** | 2  (2% / 0.3%) | 67  (53% / 1%) | 57  (45% / 48%) | 126 (2%) |
| **Total** | 545 (3%) | 15,8821 (96%) | 117 (1%) | 16,483 |

Table 3.4 shows the response patterns for “*Did (s)he have sores?”* and *“Did (s)he have an ulcer (pit) on the foot?”.*

**Table 3.4 Agreement between “*Did (s)he have sores?”* and *“Did (s)he have an ulcer (pit) on the foot?”* (adults only)**

| **Pit/ulcer on the foot** | | | |  |
| --- | --- | --- | --- | --- |
| **Did (s)he have sores?** | **Yes**  **#**  **(Row% / Col%)** | **No** | **Don’t know** | **Total** |
| **Yes** | 198 (19% / 45%) | 806 (80% / 8%) | 8 (1% / 8%) | 1,012 (9%) |
| **No** | 234 (2% / 54%) | 9,570 (97% / 91%) | 39 (0.4% / 39%) | 9,843 (90%) |
| **Don’t Know** | 1 | 87 (62% / 1%) | 52 (37% / 53%) | 140 (1%) |
| **Total** | 433 (4%) | 10,463 (95%) | 99 (1%) | 10,995 |

**QUALITATIVE OBSERVATIONS**

- Cognitive interviews suggest that the question captured the correct constructs; respondents were accurate with descriptions of both sores and ulcers. Most respondents assessed their response through direct observations of the deceased.
  - Sores were described as skin areas that have been rubbed raw, such as bedsores.
    - “I did not see any sores on her body. She just had a bit of rash on her face which was most likely because of heat.”
  - Respondents described ulcers as pit-like or sores that display as holes in the skin.
    - “The respondent understood the questions and said that only the feet for the deceased were swollen but she did not have any sores.”
- In one case in which there was a “No” response to sores or ulcers anywhere on the body but a “Yes” response pit/ulcer on the foot), it appears the respondent was not thinking of the feet as part of the body
  - *“Did (s)he have sores or ulcers anywhere on the body?”*: No, The decedent did not have any skin problems on his body. The respondent took care of the decedent's bodily hygiene and she lived with him. The respondent would accompany the decedent to doctor visits.
  - *“Did (s)he have an ulcer (pit) on the foot?”*: Yes, The last days of his life, the decedent showed signs of moderate ulcers on the foot.
- In summary, the responses captured the correct constructs and were accurate.

## 4. SWELLING, LUMP, ULCERS, PITS IN THE BREAST

**ISSUE**

There is potential confusion between the constructs of swelling or lump in the breast and ulcers (pits) in the breast. Are participants able to answer these questions? What are the response patterns by respondent characteristics? Are the response patterns different in those with greater familiarity with the deceased? Are both questions needed?

**Table 4.1 Question series asked for adult females**

| **Question** | **Comments** |
| --- | --- |
| Did she have any swelling or lump in the breast? | Required |
| Did she have any ulcers (pits) in the breast? | Required |

**QUANTITATIVE ANALYSIS**

Table 4.2 shows the response patterns and agreement for *“Did she have any swelling or lump in the breast?”* and *“Did she have any ulcers (pits) in the breast?”.*

**Table 4.2 Agreement between *“Did she have any swelling or lump in the breast?”* and *“Did she have any ulcers (pits) in the breast?”***

|  | Pits/Ulcers in the Breast | | | | | |
| --- | --- | --- | --- | --- | --- | --- |
| **Swelling/Lump in the Breast** | **Yes**  **#**  **Row%**  **Col%** | **No** | **Don’t Know** | **Refused** | **Total** |  |
| **Yes** | 39  22%  66% | 123  71%  2% | 4  2%  5% | 8  4%  60% | 174 (3%) |  |
| **No** | 20  <1%  33% | 6,068  99%  97% | 23  <1%  27% | 5  1%  40) | 6,116 (95%) |  |
| **Don’t Know** | 0 | 47  46%  1% | 55  54%  65% | 0 | 102 (2%) |  |
| **Refused** | 0 | 0 | 3 | 0 | 3 (<1%) |  |
| **Total** | 59 (1%) | 6,238 (98%) | 85 (1%) | 13 (<1%) | 6,395 |  |

Table 4.3 demonstrates characteristics of the respondent relationship to the deceased. Table 4.4 shows if the respondent lived with the deceased for the two breast questions. For the respondent relationship to the deceased, we defined “close” to include parent, child, sister, or spouse.

**Table 4.3 Response frequencies of relationship to deceased and lived with deceased**

**among deceased adult females**

| **Lived with the Deceased** | | | | |
| --- | --- | --- | --- | --- |
| **Relationship to Deceased** | **Yes**  **#**  **Row%**  **Col%** | **No** | **Don’t Know** | **Total** |
| **Close Family**** | 3,463  91%  57% | 353  9%  42% | 1  <1%  100% | 3,817 (55%) |
| **Other** | 2,604  84%  43% | 494  16%  58% | 0 | 3,098 (45%) |
| **Total** | 6,067 (88%) | 847 (12%) | 1 (<1%) | 6,915 |

** Close family defined as: sister, child, parent, spouse

**Table 4.4 Relationship of Deceased and Frequency of Responses to Female Breast Questions**

|  | **Swelling/Lump of the Breast** | | | | **Pits/Ulcers of the Breast** | | | |
| --- | --- | --- | --- | --- | --- | --- | --- | --- |
| **Relation**  **ship to deceased** | **Yes**  **#**  **Row%**  **Col%** | **No** | **Don’t Know** | **Total** | **Yes**  **#**  **Row%**  **Col%** | **No** | **Don’t Know** | **Total** |
| **Close Family**** | 88  3%  51% | 3,258  96%  53% | 55  1.6%  54% | 3,401 (53%) | 39  1.1%  66% | 3,315  97%  53% | 45  1.3%  53% | 3,399  (53%) |
| **Other** | 86  3%  49% | 2,858  95%  47% | 47  2%  46% | 2,994 (47%) | 20  0.7%  34% | 2,923  98.0%  47% | 40  1.3%  47% | 2,983 (47%) |
| **Total** | 174 (2.7%) | 6,116 (96%) | 102 (1.6%) | 6,392 | 59 (0.9%) | 6,238 (97.7%) | 85 (1.3%) | 6,382 |

** Close family defined as: sister, child, parent, spouse

Table 4.5 shows if the respondent lived with the deceased for the two breast questions

**Table 4.5 Lived with the Deceased and Frequency of Responses to Female Breast**

|  | **Swelling/Lump of the Breast** | | | | **Pits/Ulcers of the Breast** | | | |
| --- | --- | --- | --- | --- | --- | --- | --- | --- |
| **Lived with the deceased** | **Yes**  **#**  **Row%**  **Col%** | **No** | **Don’t Know** | **Total** | **Yes**  **#**  **Row%**  **Col%** | **No** | **Don’t Know** | **Total** |
| **Yes** | 152  3%  87% | 5,361  96%  88% | 78  1%  76% | 5,591 (88%) | 52  1%  88% | 5,468  98%  88% | 59  1%  69% | 5,579 (87%) |
| **No** | 22  3%  13% | 752  94%  12% | 24  3%  24% | 798 (12%) | 7  1%  12% | 767  96%  12% | 26  3%  31% | 800 (13%) |
| **Total** | 174 (2.7%) | 6,113 (95%) | 102 (1.6%) | 6,389 | 59 (1%) | 6,235 (98%) | 85 (1%) | 6,379 |

Table 4.6 demonstrates the prevalence ratios of “Don’t Know” responses (considered a non-substantive response) dependent on the respondent relationship to the deceased and if the respondent lived with the deceased.

**Table 4.6 Likelihood of responding “Don’t Know” among close family members to the deceased or respondent who lived with the deceased**

|  | **Close Family vs Other**  **PR (95% CI, p value)** | **Lived with Deceased vs Other** |
| --- | --- | --- |
| **Did she have any swelling or lump in the breast?** | 1.02  (0.7-1.5, p=0.92) | 0.51  (0.32-0.74, p<0.05) |
| **Did she have any ulcers (pits) in the breast** | 0.98  (0.7-0.1.5, p=1.00) | 1.1  (0.5 - 2.3, p = 1) |

**QUALITATIVE OBSERVATIONS**

- The sample of cognitive testing responses for questions about breast sores or pits/ulcers included 10 cases with data for each question; there was a mixture of male (4) and female (6) respondents. Half of the respondents were close family, and all but one (brother of deceased) lived with the deceased.
- Cognitive testing indicated no problems in understanding the questions. Respondents either assessed direct observation of the respondent or information from the respondent or a health professional. Respondents all understood the terms “swelling” and “lump” to indicate the presence of a mass or tumor in the breast. Respondents also demonstrated knowing that there was a difference between lumps and pits/ulcers. All responses with data to swelling and pits/ulcers were “No.” The following notes were captures from the cognitive testing interviews:
  - “The respondent understood the question and said no. I used to help bathe her and I did not feel any lump or see any swelling on the deceased's breast, she said.”
  - Respondent said his sister did not have any ulcers on her breast but bed sores. When asked how he knew that what his sister had on her breasts were bed sores and not ulcers, Respondent said ulcers do not heal fast but Deceased’s sores dried within a few days after they were cleaned with salty water on a daily basis. When asked if his sister went to the doctor after she developed these sores, Respondent said no.
  - According to the respondent, she had never had gynecological consultation for any breast problem. She never reported any pain or mass or ulcer in the breast.
- There were no “Don't Know” responses in the sample. In all 10 cases, the respondents had enough information to answer the question.

**5. OTHER FEMALE HEALTH RELATED QUESTIONS**

**ISSUE**

The following questions are reported as challenging both for interviewer and respondent:

| - Did she ever have a period or menstruate? |
| --- |
| - When she had her period, did she have vaginal bleeding in between menstrual periods? |
| - Was the bleeding excessive? |
| - Was there excessive vaginal bleeding in the week prior to death? |
| - Did her menstrual period stop naturally because of menopause or removal of uterus? |
| - At the time of death was her period overdue? |

Are participants able to answer these questions? What are the response patterns by respondent characteristics? Are the response patterns different in those with greater familiarity with the deceased? Can this question series be simplified in any way?

**Table 5.1 Question series asked for adult females**

| **Question** | **Comment** |
| --- | --- |
| Did she ever have a period or menstruate? | Required |
| When she had her period, did she have vaginal bleeding in between menstrual periods? | If “Yes” to first order question |
| Was the bleeding excessive? | If “Yes” to second order question |
| Was there excessive vaginal bleeding in the week prior to death? | If “Yes” to first order question |
| Did her menstrual period stop naturally because of menopause or removal of uterus? | If “Yes” to first order question |
| At the time of death was her period overdue? | If “No”, “Don’t Know” or “Refused” to first order question |

**QUANTITATIVE ANALYSIS**

Table 5.2 shows the response patterns for this question series.

**Table 5.2 Response patterns for female health questions**

| **Question** | **Response n, row %** | | | |  |
| --- | --- | --- | --- | --- | --- |
|  | **Yes** | **No** | **Don’t know** | **Refused** | **TOTAL** |
| Did she ever have a period or menstruate? | 4,022  63% | 2,165  34% | 198  3% | 10  <1% | 6,395  31% |
| When she had her period, did she have vaginal bleeding in between menstrual periods? | 373  9% | 2,603  65% | 1,040  26% | 6  <1% | 4,022  19% |
| Was the bleeding excessive? | 112  30% | 217  58% | 44  12% | 0 | 373  2% |
| Was there excessive vaginal bleeding in the week prior to death? | 163  4% | 3,609  90% | 245  6% | 5  <1% | 4,022  19% |
| Did her menstrual period stop naturally because of menopause or removal of uterus? | 2,085  52% | 1,673  42% | 258  6% | 6  <1% | 4,022  19% |
| At the time of death was her period overdue? | 137  7% | 1,354  67% | 516  26% | 7  <1% | 2,014  10% |
| Total |  |  |  |  | 20,848 |

Table 5.3 assesses differences in responses by respondent relationship to the deceased and if the respondent lived with the deceased. For the respondent relationship to the deceased, we defined “close” to include parent, sister, child, or spouse..

**Table 5.3 Likelihood of responding “Don’t Know” to Female Health Related Questions**

| **Question** | **Close Family vs Other**  **Prevalence Ratio**  **(95%CI, p value)** | **Lived with Deceased vs**  **Other** |
| --- | --- | --- |
| Did she ever have a period or menstruate? | 0.81  (0.6-1.1, p= 0.13) | 0.48  (0.35-0.65, p<0.001) |
| When she had her period, did she have vaginal bleeding in between menstrual periods? | 0.71  (0.64-0.80, p<0.001) | 0.77  (0.67-0.89, p<0.001) |
| Was the bleeding excessive? | 1.31  (0.7-2.4, p=0.33) | 0.56  (0.28-1.11, p=0.1) |
| Was there excessive vaginal bleeding in the week prior to death? | 1.23  (0.9-1.5, p=0.5) | 0.54  (0.40-0.72, p<0.001) |
| Did her menstrual period stop naturally because of menopause or removal of uterus? | 1.12  (0.9-1.4, p=0.5) | 0.48  (0.36-0.64,p<0.001) |
| At the time of death was her period overdue? | 0.74  (0.61-0.9, p<0.05) | 0.70  (0.58-0.84, p<0.001) |

**QUALITATIVE OBSERVATIONS**

- The sample of cognitive testing responses for these questions included 11 total respondents with 7 female and 4 male respondents. All respondents were either close family (5) or extended family (6) who lived with the deceased.
- The questions captured the correct constructs. All respondents considered the deceased’s history of menstruation when answering the questions.
  - There were three ways in which respondents assessed their answer:
    - Direct observation: either from discussions with the deceased or physical observation
      - “Respondent understood the question and said yes. Deceased used to have her periods with minimal to less bleeding for 3 days.”
    - Indirect observation: hearing from a doctor or others
      - “The deceased was married but had never had children. The respondent was the niece of the decedent; to her knowledge the decedent had no problems related to menstruation.”
    - Logic: assessment based on observations of associated indicators such as age, children, etc.
      - “[‘Deceased] was in menopause,’ said respondent.”
  - For “Don’t Know” answers, respondents did not have adequate information for a valid response
    - “The respondent had no information on the deceased's gynecological problems. The deceased was very reserved.”
- Unlike knowledge about breast lumps and ulcers, the insight on menstruation is gendered. Women were more likely to assess their responses through direct and indirect observations, and men were more likely to answer “Don't Know.” However, not all questions required direct observations, and the validity of each should be considered separately.
  - “*Did she ever have a period or menstruate?”:* Men and women considered this question using logic (e.g., the deceased had children) and indirect observations. Only one male answered “Don’t Know” for his Aunt. Answers were accurate.
  - “*When she had her period, did she have vaginal bleeding in between menstrual periods?”:* Responses to this question were gendered. All men and a few women answered, “Don’t Know.” Only three women were able to give substantive answers, all of which were “No.”
  - *“Was there excessive vaginal bleeding in the week prior to death?”:* Men and women gave substantive answers; only one male answered, “Don’t Know.”
  - *“Did her menstrual period stop naturally because of menopause or removal of uterus?”:*Men and women gave substantive answers; only one male answered, “Don’t Know.”
- In summary, knowledge about menstruation is gendered, but limited to close family members. Most males, and several females were therefore unable to answer questions that required intimate knowledge about the deceased’s history of menstruation and vaginal bleeding. Other questions, such as “*Did she ever have a period or menstruate?”* and “*Did her menstrual period stop naturally because of menopause or removal of uterus?”* did not require more intimate knowledge; both men and women could answer the questions based on indirect knowledge and logic. All substantial answers (Yes or No) given to all questions were accurate.

## 6. DIAGNOSIS BY A HEALTH PROFESSIONAL VS SYMPTOM REPORT

**ISSUE**

Measurement error is more likely with questions on diagnosis than with questions on symptoms. The following summary is the same for each of the “diagnosis” questions tested: dengue fever, COPD, and dementia. A review of the accuracy and consistency in responses patterns for medical diagnoses and their related symptoms can help inform the appropriate selection of questions.

**DIAGNOSIS QUESTION SERIES**

The diagnosis questions included in the medical history section of the 2016 WHO VA questionnaire are listed in the table below.

**Table 6.1 Diagnosis question series for children and adults**

| **Medical history, diagnosis question list** |
| --- |
| Was there any diagnosis by a health professional of tuberculosis? |
| Was an HIV test ever positive? |
| Was there any diagnosis by a health professional of AIDS? |
| Did (s)he have a recent positive test by a health professional for malaria? |
| Did (s)he have a recent negative test by a health professional for malaria? |
| Was there any diagnosis by a health professional of **dengue fever?** |
| Was there any diagnosis by a health professional of **measles?** |
| Was there any diagnosis by a health professional of high blood pressure? |
| Was there any diagnosis by a health professional of heart disease? |
| Was there any diagnosis by a health professional of diabetes? |
| Was there any diagnosis by a health professional of asthma? |
| Was there any diagnosis by a health professional of epilepsy? |
| Was there any diagnosis by a health professional of cancer? |
| Was there any diagnosis by a health professional of **Chronic Obstructive Pulmonary Disease (COPD)**? |
| Was there any diagnosis by a health professional of **dementia**? |
| Was there any diagnosis by a health professional of depression? |
| Was there any diagnosis by a health professional of **stroke**? |
| Was there any diagnosis by a health professional of sickle cell disease? |
| Was there any diagnosis by a health professional of kidney disease? |
| Was there any diagnosis by a health professional of liver disease? |

General Qualitative Observations

There were two general patterns in which respondents evaluated the “health conditions” of the deceased: A) Medical diagnoses from health professional or B) Symptoms perceived to be related to the condition, as shown in the following figure:

|  |  | A1: Affirmative Diagnosis of condition |
| --- | --- | --- |
|  | A: Medical Diagnosis of condition | A2: Diagnosis of another condition |
| Basis for Response to Diagnosis Question |  |  |
|  | B:Symptoms perceived to be related to condition |  |

In **response pattern A:** respondents considered the known medical diagnoses of the deceased. This pattern resulted in accurate responses when the respondent (1) knew the correct diagnosis and (2) knew the differences between the diagnosis and each other item listed. In other words, the respondent must know that the deceased was diagnosed with dengue fever, and that dengue fever is distinct from yellow fever, malaria, etc. Response errors resulted when these two conditions were not met.

In all cognitive interviews on Dengue, the only five “Yes” respondents (all from Zambia) were **false positives**.

One of these respondents **did not know the correct diagnosis**. He explained that the deceased had a fever and tested negative for malaria. When he heard “dengue fever” listed on the survey, he figured that “dengue” must have been the name of his friend’s condition, even though he had never heard of it before. When probed about the symptoms related to dengue—such as a rash—the respondent said that his friend did not have these.

Another respondent **did not know the differences between dengue and other conditions.** He answered “Yes” because he figured dengue fever was the result of malaria, which his father had been diagnosed with.

In the **response pattern B**, respondents did not have an official diagnosis; they considered symptoms perceived to be associated with the condition. Since documentation of diagnosis is not available to confirm the response, it is impossible to know if there was a response error in terms of “false positives.” It was only clear that there was a response error when the respondent described symptoms that are not actually associated with the condition.

Possible response errors occurred when responses assumed symptoms were associated with the item. In other words, respondents said “Yes” to COPD without any official diagnosis because respondents displayed associated symptoms such as shortness of breath.

o Two Moroccan respondents—one reporting as a father and the other reporting as a husband —based their “Yes” responses on the fact that the deceased had noticeable breathing issues in the period immediately preceding their death, and not on whether or not they knew about an affirmative diagnosis of COPD.

§ *She [the wife] had very short/quick breathing, especially at night, during the last 15 days before her death.*

§ *The deceased suffered from seizures since his birth, and he was under treatment and monitored by a neurologist. He was hospitalized 1.5 months before his death and suffered from respiratory distress 20 days before his death. The deceased was in intensive care and was on artificial respiration in the last days of his life.*

Response error was clearer when the respondent did not correctly associated symptoms with COPD.

o False Positive: *The respondent did not understand what COPD involves. As he mentioned that since blood samples were collected from the deceased for tests, he assumed that COPD is verified through the blood.*

Due to the relative familiarity with tuberculosis, HIV, and malaria, no analyses were conducted for these conditions. Analyses are provided for dengue, measles, COPD, dementia and stroke in this section. Observations are made from two datasets -- 1) the Primary dataset, which is used to compare response patterns between the diagnosis and key associated symptoms for each diagnosis, and 2) the reference dataset, where causes of death were assigned by PCVA for the for the diagnostic condition of interest. The analysis includes response patterns for the diagnosis and related symptoms as follows:

1) Frequency of responses to diagnosis question from all respondents and from those assigned a cause associated with the condition in question; and

2) Key symptoms questions in those assigned a cause associated with the condition in question (or where no cause was assigned, among those for whom a “yes” response was reported to the diagnosis question). Key symptoms were selected based on inclusion of the symptom in the cause of death assignment by physician certification of VA (PCVA) or by the automated algorithms.

1. **Dengue**

**Table 6A.1 Dengue-related diagnosis and symptom questions (children and adults)**

| **Question** | **Comment** |
| --- | --- |
| Was there any diagnosis by a health professional of dengue fever? | Required |
| For how long was (s)he ill before death?  Enter how many days  Enter how many months | Required (option: days,months,years) |
| Did (s)he have a fever | Required |
| How long did the fever last?  Enter how many days  Enter how many months | If “yes” to first order question (option: days, months, years) |
| Did (s)he vomit? | Required |
| Did (s)he have a severe headache? | Required |
| During the illness that led to death, did (s)he bleed from anywhere? | If “yes” to first order question |
| Did (s)he bleed from the nose, mouth or anus? | If “yes” to first order question |

Using the Primary dataset, Table 6A.2 explores the frequency of dengue fever diagnosis and the symptoms reported by those for whom a “yes” response was given for dengue fever diagnosis.

**Table 6A.2 Dengue fever diagnosis and symptom frequencies (From the Primary Dataset)**

| **Question** | **Yes (%)** | **No (%)** | **DK (%)** | **Total** |
| --- | --- | --- | --- | --- |
| Was there any diagnosis by a health professional of dengue fever? | 102 (1%) | 11,586 (74%) | 3,894 (25%) | 15,552 |
| Among those who reported dengue fever diagnosis: | | | | |
| For how long was (s)he ill before death?  (less than 3 weeks) | 37 (36%) | 65 (64%) | 0 | 102 |
| Did (s)he have a fever? | 71 (70%) | 31 (30%) | 0 | 102 |
| How long did the fever last?  (1-2 weeks)   - among those reporting fever - among those who reported dengue fever diagnosis | 16 (22%)  16 (16%) | 55 (78%)  86 (84%) | 0  0 | 71  102 |
| Did (s)he vomit? | 43 (42%) | 59 (58%) | 0 | 102 |
| Did (s)he have a severe headache? | 40 (39%) | 51 (50%) | 11 (11%) | 102 |
| During the illness that led to death, did (s)he bleed from anywhere? | 9 (9%) | 93 (91%) | 0 | 102 |
| Did (s)he bleed from the nose, mouth or anus?   - among those reporting bleeding - among those who reported dengue fever diagnosis | 7 (78%)  7 (7%) | 2 (22%)  95 (93%) | 0  0 | 9  102 |

Using the reference dataset with PCVA-assigned cause of death, Table 6A.3 explores the frequency of dengue fever diagnosis by healthcare professionals among all deceased and those assigned dengue fever as a cause of death.

**Table 6A.3 Dengue fever diagnosis and symptom response frequencies among all respondents and those assigned dengue fever as a cause of death (COD) (From the Reference Data Set)**

| Question | **All deceased**  **(n=5,284 )** | | | | |  | **Those assigned dengue fever as COD by PCVA**  **(n=46 )** | | | |
| --- | --- | --- | --- | --- | --- | --- | --- | --- | --- | --- |
|  | **Yes**  **(%)** | **No**  **(%)** | **Ref** | **DK (%)** | **Total** |  | **Yes**  **(%)** | **No**  **(%)** | **DK**  **(%)** | **Total** |
| Was there any diagnosis by a health professional of dengue fever? | 22  (1%) | 5,203  (98%) | 2  (<1%) | 57  (1%) | 5,284 |  | 1  (2%) | 45  (98%) | 0 | 46 |
| Among those who reported dengue fever diagnosis: | | | | | |  | Among those assigned dengue fever as a COD*: | | | |
| For how long was (s)he ill before death?  (less than 3 weeks) | 7  (32%) | 15  (68%) | 0 | 0 | 22 |  | 16  (35%) | 30  (65%) | 0 | 46 |
| Did (s)he have a fever? | 7  (32%) | 15  (68%) | 0 | 0 | 22 |  | 10  (22%) | 35  (76%) | 1  (2%) | 46 |
| How long did the fever last?  (1-2 weeks)   - among those reporting fever - among those who reported dengue fever diagnosis | 3  (42%)  3  (14%) | 4  (58%)  19  (86%) | 0  0 | 0  0 | 7  22 |  | 9  (90%) | 1  (10%) | 0 | 10  N/A |
| Did (s)he vomit? | 6  (27%) | 16  (73%) | 0 | 0 | 22 |  | 19  (41%) | 27  (59%) | 0 | 46 |
| Did (s)he have a severe headache? | 6  (27%) | 16  (73%) | 0 | 0 | 22 |  | 4  (9%) | 41  (89%) | 1  (2%) | 46 |
| During the illness that led to death, did (s)he bleed from anywhere? | 2  (9%) | 20  (91%) | 0 | 0 | 22 |  | 0 | 46  (100%) | 0 | 46 |
| Did (s)he bleed from the nose, mouth or anus?   - among those reporting bleeding - among those who reported dengue fever diagnosis | 1  (50%)  1  (4%) | 1  (50%)  21  (96%) | 0  0 | 0  0 | 2  22 |  |  |  |  | N/A |

*We would have liked to have shown response patterns to corresponding dengue fever symptoms among those that responded “yes” to dengue fever diagnosed by a health professional; however, given that only 1 respondent indicated yes, the analysis of symptom response patterns is among all who were assigned dengue fever as a COD.

**QUALITATIVE OBSERVATIONS**

- Cognitive testing showed false positives resulted from confusing dengue with other illnesses (Pattern A and Pattern B).
- Some respondents confused or conflated the disease under question with another condition—so even though they were answering based on whether or not the deceased had been given a diagnosis, they were thinking about the wrong disease. This was particularly common in the question about dengue fever, where respondents were equating dengue and yellow fever.
- Confusing and conflating dengue fever with other diseases—primarily yellow fever, malaria, and sickle cell anemia—was common, and in a few cases led to false positives; across all of the cognitive interviews, only five respondents answered “Yes”.
- All of these appear to be false positives. For example, one respondent who was answering about a friend and answered “Yes” explained that the deceased had a fever. Upon further probing the respondent explained that the decedent had a negative malaria test, so he figured that dengue fever was the name of the fever that his friend suffered from at the end of his life, although he also admitted to not knowing what dengue fever was. However, when asked about other symptoms related to dengue, the respondent said that his friend did not have these. Similarly, another respondent who answered “Yes” appeared to think that dengue fever was the result of malaria, which his father had been diagnosed with.

1. **Measles**

**Table 6B.1 Measles-related questions, for children and adults**

| **Question** | **Comment** |
| --- | --- |
| Was there any diagnosis by a health professional of measles? | Required |
| For how many days was (s)he ill before death? | Required |
| Did (s)he have a fever? | Required |
| How long did the fever last? | If “yes” to first order question |
| Did the fever continue until death? | If “yes” to first order question |
| Did (s)he have a cough? | Required |
| During the illness that led to death, did (s)he have any skin rash? | Required |
| Where was the rash? | If “yes” to first order question |
| Did (s)he have measles rash (use local term)? | If “yes” to first order question |

Using primary dataset, Table 6B.2 shows measles symptom frequencies among those who reported “Yes” to measles diagnosis.

**Table 6B.2 Measles diagnosis and symptom frequencies (From the Primary Dataset)**

| **Question** | **Yes (%)** | **No (%)** | **DK (%)** | **Refused** | **Total** |
| --- | --- | --- | --- | --- | --- |
| Was there any diagnosis by a health professional of measles? | 77 (1%) | 11,627 (74%) | 3,883 (25%) | 5  (<1%) | 15,592 |
| Among those who reported measles diagnosis: | | | | | |
| For how many days was (s)he ill before death?  (less than 3 weeks) | 22 (29%) | 55 (71%) | 0 | 0 | 77 |
| Did (s)he have a fever? | 50 (65%) | 26 (35%) | 1 | 0 | 77 |
| How long did the fever last?  (less than a week)   - among those reporting fever - among those who reported measles diagnosis | 27 (54%)  27 (35%) | 23 (46%)  50 (65%) | 0  0 | 0  0 | 50  77 |
| Did the fever continue until death?   - among those reporting fever - among those who reported measles diagnosis | 35 (70%)  35 (46%) | 14 (28%)  41 (53%) | 1 (2%)  1(1%) | 0  0 | 50  77 |
| Did (s)he have a cough? | 43 (56%) | 33 (43%) | 1 (1%) | 0 | 77 |
| During the illness that led to death, did (s)he have any skin rash? | 14 (19%) | 62 (81%) | 1 | 0 | 77 |
| For how many days did (s)he have the skin rash?  (3 or more days per PCVA)   - among those reporting a rash - among those who reported measles diagnosis | 7 (50%)  7 (9%) | 7 (50%)  70 (91%) | 0  0 | 0  0 | 14  77 |
| Where was the rash?   - among those reporting a rash - among those who reported measles diagnosis | 1 (7%)  1 (1%) | 13 (93%)  76 (99%) | 0  0 | 0  0 | 14  77 |
| Did (s)he have measles rash (use local term)?   - among those reporting a rash - among those who reported measles diagnosis | 10 (72%)  10 (13%) | 3 (21%)  67 (86%) | 1 (7%)  1 (1%) | 0  0 | 14  77 |

** There were no measles deaths in the reference dataset for further analysis.

1. **COPD**

**Table 6C.1 COPD-related questions, for adults**

| **Question** | **Comment** |
| --- | --- |
| Was there any diagnosis by a health professional of COPD? | Required |
| Did (s)he have a cough? | Required |
| For how many days did she have a cough? (at least 3 weeks) | If “yes” to first order question |
| Was the cough productive, with sputum? | If “yes” to first order question |
| Did (s)he have breathlessness? | Required |
| Did (s)he have wheezing? | Required |

Using the Primary dataset, Table 6C.2 explores the frequency of COPD diagnosis and the symptoms reported by those for whom a “Yes” response was given for COPD diagnosis in.

**6C.2 COPD diagnosis and symptom frequencies (From the Primary Dataset)**

| Question | **Yes (%)** | **No (%)** | **DK (%)** | **Total** |
| --- | --- | --- | --- | --- |
| Was there any diagnosis by a health professional of COPD? | 531 (4%) | 9,274 (72%) | 3,148 (24%) | 12,953 |
| Among those who reported COPD diagnosis: | | | | |
| Did (s)he have a cough? | 327 (62%) | 203 (38%) | 1 (<1%) | 531 |
| For how many days did she have a cough? (at least 3 weeks)   - among those reporting a cough - among those who reported COPD diagnosis | 127 (39%)  127 (24%) | 200 (61%)  404 (76%) | 0  0 | 327  531 |
| Was the cough productive, with sputum?   - among those reporting a cough - among those who reported COPD diagnosis | 185 (57%)  185 (35%) | 140 (43%)  344 (65%) | 2 (<1%)  2 (<1%) | 327  531 |
| Did (s)he have breathlessness? | 387 (73%) | 143 (27%) | 1 (<1%) | 531 |
| Did (s)he have wheezing? | 276 (52%) | 253 (48%) | 2 (<1%) | 531 |

Using the reference dataset with PCVA-assigned cause of death, Table 6C.3, explores the frequency of “Yes” responses to COPD diagnosis by healthcare professionals among all deceased and those assigned COPD as a cause of death.

**Table 6C.3 COPD diagnosis and symptom response frequencies among all respondents and those assigned COPD as a COD (From the Reference Dataset)**

| Question | **All deceased**  **(n=5,094 )** | | | |  | **Those assigned COPD as COD by PCVA**  **(n=67)** | | | |
| --- | --- | --- | --- | --- | --- | --- | --- | --- | --- |
|  | **Yes**  **(%)** | **No**  **(%)** | **DK (%)** | **Total** |  | **Yes**  **(%)** | **No**  **(%)** | **DK**  **(%)** | **Total** |
| Was there any diagnosis by a health professional of COPD? | 196  (4%) | 4,825  (95%) | 73  (1%) | 5,094 |  | 19  (28%) | 46 (69%) | 2  (3%) | 67 |
| Among those who reported COPD diagnosis: | | | | | | | | | |
| Did (s)he have a cough? | 112  (57%) | 84  (43%) | 0 | 196 |  | 13  (69%) | 6  (31%) | 0 | 19 |
| For how many days did she have a cough? (at least 3 weeks)   - among those reporting a cough - among those who reported COPD diagnosis | 32  (29%)  32  (16%) | 64  (57%)  148  (76%) | 16  (14%)  16  (8%) | 112  196 |  | 9  (69%)  9  (47%) | 4  (31%)  10  (53%) | 0  0 | 13  19 |
| Was the cough productive, with sputum?   - among those reporting a cough - among those who reported COPD diagnosis | 64  (57%)  64  (33%) | 47  (42%)  131  (67%) | 1  (1%)  1  (<1%) | 112  196 |  | 7  (54%)  7  (37%) | 6  (46%)  12  (63%) | 0  0 | 13  19 |
| Did (s)he have breathlessness? | 76  (39%) | 119  (61%) | 1  (<1%) | 196 |  | 10  (53%) | 7  (37%) | 2  (10%) | 19 |
| Did (s)he have wheezing? | 61  (31%) | 126  (64%) | 9  (5%) | 196 |  | 4  (21%) | 14  (74%) | 1  (5%) | 19 |

**QUALITATIVE OBSERVATIONS**

Recent cognitive testing of the medical diagnosis questions found evidence of false positive response errors. From cognitive testing results:

- *“ In one response pattern, respondents only considered symptoms they believed to be associated with the condition.”*
- *“Other respondents based their answer on whether or not the decedent displayed any symptoms they understood to be related to COPD. “*
- *“ In some cases, the respondents at least knew about some of the symptoms associated with the condition. In other cases, the respondents did not know the condition or the symptoms, but still gave a Yes/No response. These were the situations that were mostly likely to produce response errors”*
- *“Both of these respondents appeared to comprehend COPD as a respiratory disease and based their answer not on the fact that they had heard about an actual diagnosis of COPD, but rather on the fact that the decedent had lung Issues”*

From the cognitive testing results, there is concern that many of these affirmative answers to the COPD diagnosis question are false positives. Can we interpret findings in the quantitative analyses similarly i.e. the fact that 25-35% of respondents who reported a diagnosis of COPD did not report any of the common COPD symptoms (cough, wheeze, breathlessness)? Does this support our assumption that many of these are false positives and indicate a poor understanding of the medical term?

1. **Dementia**

**Table 6D.1 Dementia-related questions, for adults**

| **Question** | **Comment** |
| --- | --- |
| Was there any diagnosis by a health professional of dementia? | Required |
| Did (s)he have mental confusion? | Required |

Using the primary dataset, Table 6D.2 explores the frequency of dementia diagnosis and the symptoms reported by those for whom a “Yes” response was given for dementia diagnosis. To note, dementia is not included as a cause of death in WHO’s VA cause list.

**Table 6D.2 Dementia diagnosis and symptom frequencies (From the Primary Dataset)**

| Question | **Yes (%)** | **No (%)** | **DK (%)** | **Total** |
| --- | --- | --- | --- | --- |
| Was there any diagnosis by a health professional of dementia? | 493 (4%) | 9,256 (71%) | 3,204 (25%) | 12,953 |
| Among those who reported dementia diagnosis: | | | | |
| Did (s)he have mental confusion? | 79 (16%) | 414 (84%) | 0 | 493 |

**QUALITATIVE OBSERVATIONS**

False positives resulted from associating any mental health issue with dementia (Pattern B)

*[The deceased] was never diagnosed with dementia. It sounds like he did have mental health issues, but the Respondent does not know if he was diagnosed with anything. Also, there may have been confusion about differences between dementia and confusion because the word is difficult to translate in Nyanja. The respondent confused memory loss with mental confusion, as he said the deceased used to wake up in the night asking and looking for the knife and he wanted to cause violence in that he was saying he wanted to stab someone. When asked if the deceased was diagnosed with mental confusion by a health professional, the respondent said he would not really know if the deceased was diagnosed with the mental problem by a health professional because he only stayed with the deceased for two years and the that problem of waking up trying to look for a knife to stab someone had started even earlier when the deceased used to stay with his sister. After that the respondent was given examples of what dementia is, he was asked if he thinks the deceased had dementia he said yes because the deceased could forget people’s names. The conclusion is that the deceased might have had both dementia and mental confusion, even if the respondent failed to distinguish the two concepts.*

1. **Stroke**

**Table 6E.1 Stroke-related questions, for adults**

| **Question** | **Comment** |
| --- | --- |
| Was there any diagnosis by a health professional of stroke? | Required |
| During the illness that led to death, did (s)he have a severe headache? | Required |
| Was (s)he unconscious during the illness that led to death? | Required |
| How long before death did unconsciousness start? | Required |
| Was (s)he in any way paralysed? | Required |
| Did (s)he have paralysis of only one side of the body? | If “yes” first order question |
| Did (s)he have difficulty swallowing? | Required |
| Did (s)he have difficulty with swallowing liquids? | If “yes” to first order question |

Using the Primary dataset, Table 6E.2 explores the frequency of Stroke diagnosis and the symptoms reported by those for whom a “Yes” response was given for stroke diagnosis question.

**Table 6E.2 Stroke diagnosis and symptom frequencies (From the Primary Dataset)**

| Question | **Yes (%)** | **No (%)** | **DK (%)** | **Total** |
| --- | --- | --- | --- | --- |
| Was there any diagnosis by a health professional of stroke? | 945 (7%) | 8,942 (69%) | 3,066 (24%) | 12,953 |
| Among those who reported a stroke diagnosis: | | | | |
| During the illness that led to death, did (s)he have a severe headache? | 337 (36%) | 573 (60%) | 35 (4%) | 945 |
| Was (s)he unconscious during the illness that led to death? | 263 (29%) | 669 (70%) | 13 (1%) | 945 |
| How long before death did unconsciousness start? (at least 6 hours)   - among those reporting unconsciousness - among those who reported stroke diagnosis | 176 (67%)  176 (19%) | 84 (32%)  766 (81%) | 3 (1%)  3 (<1%) | 263  945 |
| Was (s)he in any way paralysed? | 593 (63%) | 344 (36%) | 8 (1%) | 945 |
| Did (s)he have paralysis of only one side of the body?   - among those reporting paralysis - among those who reported stroke diagnosis | 502 (85%)  502 (53%) | 91 (15%)  443 (47%) | 0  0 | 593  945 |

Using the reference dataset with PCVA-assigned cause of death, Table 6E.3 explores the frequency of stroke diagnosis by healthcare professionals, among all deceased and those assigned Stroke as a cause of death.

**Table 6E.3 Stroke diagnosis and symptom response frequencies among all respondents and those assigned Stroke as a COD (From the Reference Dataset)**

| Question | **All deceased**  **(n=5,094)** | | | | |  | **Those assigned Stroke as COD by PCVA**  **(n=303 )** | | | |
| --- | --- | --- | --- | --- | --- | --- | --- | --- | --- | --- |
|  | **Yes**  **%** | **No**  **%** | **DK %** | **Refused**  **%** | **Total** |  | **Yes**  **%** | **No**  **%** | **DK**  **%** | **Total** |
| Was there any diagnosis by a health professional of stroke? | 438  (9%) | 4,599 (90%) | 54 (1%) | 3  (<1%) | 5,094 |  | 214 (71%) | 86 (28%) | 3 (1%) | 303 |
| Among those who reported stroke diagnosis: | | | | | | | | | | |
| During the illness that led to death, did (s)he have a severe headache? | 137 (31%) | 291 (67%) | 10  (2%) | 0 | 438 |  | 55  (26%) | 153 (71%) | 6  (3%) | 214 |
| Was (s)he unconscious during the illness that led to death? | 55  (13%) | 374  (85%) | 9  (2%) | 0 | 438 |  | 21  (10%) | 187  (87%) | 6  (3%) | 214 |
| How long before death did unconsciousness start? (at least 6 hours before death)   - among those reporting unconsciousness - among those who reported stroke dx | 25 (45%)  25  (6%) | 29 (53%)  412  (94%) | 0  0 | 1  (2%)  1  (<1%) | 55  438 |  | 0  0 | 21 (100%)  214  (100%) | 0  0 | 21  214 |
| Was (s)he in any way paralysed? | 180  (41%) | 251  (57%) | 7  (2%) | 0 | 438 |  | 101  (48%) | 108  (50%) | 5  (2%) | 214 |
| Did (s)he have paralysis of only one side of the body?   - among those reporting paralysis - among those who reported stroke dx | 137 (76%)  137  (31%) | 43 (24%)  301  (69%) | 0  0 | 0  0 | 180  438 |  | 74  (73%)  74  (35%) | 27 (27%)  140  (65%) | 0  0 | 101  214 |

## 7. VACCINATIONS

**Issue**

The question “*Select EPI vaccines done”* is complicated, as it requires the interviewer to know what the complete vaccine schedule is for their country and to assess the vaccination card for completion. We expect there is much room for error here, and we want to optimize the question structure, simplify data collection, and provide appropriate field training guidance to ensure quality of this information.

Also, documentation of vaccine status is required for a response to *“Select EPI vaccines done”*; a concern has been reported that for many respondents, this documentation may not be available, because it was thrown away, buried with the child, or otherwise lost.

**Table 7.1 Question series, asked for neonates (not stillbirths) and children**

| **Question** | **Comment** |
| --- | --- |
| Did (s)he receive any immunizations? | Required |
| Do you have the child's vaccination card? | If “yes” to first order question |
| Can I see the vaccination card (note the vaccines the child received)? | If “yes” to second order question |
| Select EPI vaccines done | If “yes” to third order question |

**QUANTITATIVE ANALYSIS**

There were 4,492 neonates and children eligible to be asked the immunization series of questions. Of these, a response documenting immunization was received was provided for 15% (n=656) respondents

Table 7.2 shows the response patterns for the immunization question series, for respondents that followed the standard skip pattern.

**Table 7.2 Frequency of responses to immunization question series**

| **Question** | **Yes (%)** | **No (%)** | **Ref (%)** | **DK (%)** | **Total** |
| --- | --- | --- | --- | --- | --- |
| Did (s)he receive any immunizations? | 1,776 (63%) | 805 (28%) | 1 (<1%) | 242 (9%) | 2,824 |
| Do you have the child's vaccination card? | 796 (45%) | 974 (55%) | 1 (<1%) | 5 (<1%) | 1,776 |
| Can I see the vaccination card (note the vaccines the child received)? | 528 (84%) | 96 (15%) | 0 | 6 (1%) | 796** |
| Select EPI vaccines done | 656 (responses*** | 2 | N/A | N/A | 658 |

* Of the total number of children and neonates that were asked about immunization (n=2,824)

**Includes 166 respondents that left this item blank (presumably as part of a different programmed skip pattern).

*** Of these, 14 responses did not list specific vaccines; only “received” or “up to date until x age”, 78 respondents who did not respond to *“Do you have the child's vaccination card?”* provided vaccine information

**QUALITATIVE OBSERVATIONS**

- Unlike the other questions included in the cognitive interview, this question was not probed—cognitive interviewers were instead instructed to observe the verbal autopsy and determine whether or not the respondent was using the decedent’s vaccine card (i.e. their record of vaccines given by a healthcare provider) or their memory when answering the question. The validity of this question relies on the fact that respondents are using a memory aid, such as a vaccine card; however, this assumption has been called into question as the verbal autopsy instrument has been evaluated over the past decade.
- Only seven out of the 49 respondents who received this question “*Do you have the child's vaccination card?”* used their vaccine cards to answer it; the rest either indicated that they did not know (n=13) or based their response on memory (n=29).

## 8. INJURY QUESTIONS

**ISSUE**

Feedback from the field indicates significant frustration by interviewers and respondents in completing the remainder of the long questionnaire when somebody who was not otherwise ill clearly died of an accident. Specifically, if they have answered “Yes” to “*Did (s)he suffer from any injury or accident that led to her/his death?”,* after answering the remainder of the injury series, should the respondent continue through all of the remaining questions of the questionnaire? Some reasons to ask subsequent questions after indication of death by injury include to determine if the death was maternal related or to determine if the injury was caused by an underlying medical condition.

**QUANTITATIVE ANALYSIS**

**A.**

Table 8.1 shows the frequency of responses to “*Did (s)he suffer from any injury or accident that led to her/his death?”* from the Primary Dataset

**Table 8.1 Injury question response frequency, among neonates, children, and adults (Primary dataset)**

| **Question** | **Yes (%)** | **No (%)** | **Ref (%)** | **DK (%)** | **Total** |
| --- | --- | --- | --- | --- | --- |
| Did (s)he suffer from any injury or accident that led to her/his death? | 1,919 (10%) | 16,471 (89%) | 2 (<1%) | 53 (<1%) | 18,445 |

**B.**

There are approximately 25 first order (required) symptom questions in the standard adult WHO questionnaire (for example, cough, fever, vomiting). Table 8.3 explores the frequency of respondents reporting affirmative responses to these 25 first order symptom questions among those who reported an injury (Defined as a “Yes” response to “*Did (s)he suffer from any injury or accident that led to her/his death*?”)

**Table 8.3 Number of symptoms reported (out of 25) among those reporting an injury**

**(Primary dataset)**

| **Number of Symptom Questions with a “Yes” Response** | **n (%)** |
| --- | --- |
| **0** | 171 (9%) |
| **1** | 279 (15%) |
| **2 - 4** | 1,169 (61%) |
| **5 - 6** | 184 (10%) |
| **> 6** | 116 (5%) |
| **Total** | 1,919 |

**C.**

**Reference Study Analysis**

Tables 8.4-8.10 use the referent data from physician certified verbal autopsy (PCVA) results in the reference dataset to observe response patterns among those for whom an injury-related death was assigned as the underlying cause of death (UCOD). Injury-related UCOD codes included: V01 to Y98 (*Note there were no S or T code UCOD in the SA dataset).

**Table 8.4 Injury question response frequency (Reference dataset)**

| **Question** | **Yes (%)** | **No (%)** | **DK (%)** | **Total** |
| --- | --- | --- | --- | --- |
| **Did (s)he suffer from any injury or accident that led to her/his death?”** | 680 (13%) | 4,699 (87%) | 9 (<1%) | 5,388 |

**Table 8.5 Response Frequencies of “*Did (s)he suffer from any injury or accident that led to her/his death?”* among those assigned INJURY and NON-INJURY UCOD**

| **Did (s)he suffer from any injury or accident that led to her/his death?”** | **Injury UCOD** | **Non-Injury UCOD** | **R99 (Ill-defined and unknown)** | **Total** |
| --- | --- | --- | --- | --- |
| **Yes** | 582 (85%) | 79 (12%) | 19 (3%) | 680 (13%) |
| **No** | 102 (2%) | 4,178 (89%) | 419 (9%) | 4,699 (87%) |
| **DK** | 1 (11%) | 8 (89%) | 0 | 9 (<1%) |
| **Total** | 685 (13%) | 4,265 (79%) | 438 (8%) | 5,388 |

**Table 8.6 Mean number of affirmative responses to first order symptoms questions in the WHO questionnaire among those assigned an injury and a non-injury UCOD**

| **UCOD** | **Mean # of Affirmative Responses to the 25 First order Symptom Questions**  **Median (IQR)** | **Total** |
| --- | --- | --- |
| **Injury** | 0.8 (0-1) | 685 |
| **Non-Injury** | 3 (1-4) | 4,265 |

**Table 8.7 Non-injury underlying cases of death among those where the respondent indicated the presence of an injury (**Defined as a “Yes” response to “*Did (s)he suffer from any injury or accident that led to her/his death*?”**) (n=79)**

| **UCOD (ICD block)** | **n (%)** |
| --- | --- |
| Infectious/Parasitic (A/B) | 27 (34%) |
| Circulatory (I) | 22 (28%) |
| Neoplasm (C) | 9 (11%) |
| Digestive (K) | 5 (6%) |
| Respiratory (J) | 4 (5%) |
| Endocrine/Metabolic (E) | 3 (4%) |
| Nervous System (G) | 3 (4%) |
| Mental/Behavioral (F) | 3 (4%) |
| Pregnancy (O) | 1 (1%) |
| Other NCD | 2 (3%) |

##### QUALITATIVE OBSERVATIONS

The injury series was not evaluated with the cognitive interviews

**9. URINE**

**ISSUE**

In this series, the following three questions are skipped if NO/DK/Ref was answered to “*Did (s)he have any urine problems?”*

- - Did (s)he go to urinate more often than usual?
  - Did (s)he stop urinating?
  - During the final illness did (s)he ever pass blood in the urine?

What is the consistency between a “Yes” response to “*Did (s)he have any urine problems?”*

and a “Yes” response to the subsequent questions? Inconsistencies would flag potential false positives; respondent may not know what urine problems are (e.g., blood in pee)?

**Table 9.1 Question series, asked for children and adults**

| **Question** | **Comment** |
| --- | --- |
| Did (s)he have any urine problems? | Required |
| Did (s)he stop urinating? | If “Yes” to first order question |
| Did (s)he go to urinate more often than usual? | If “Yes” to first order question |
| During the final illness did (s)he ever pass blood in the urine? | If “Yes” to first order question |

**QUANTITATIVE ANALYSIS**

Table 9.2 summarizes the response frequencies of the first order question and follow up questions.

**Table 9.2. Response Frequencies to Urine Questions**

| **Question** | **Yes** | **No** | **DK** | **Refused** | **Total** |
| --- | --- | --- | --- | --- | --- |
| Did (s)he have any urine problems? | 2,092 (13%) | 14,170 (85%) | 392  (2%) | 4  (<1%) | 16,658 |

| Of those who answered “Yes” to a urine problem: | | | | | |  |
| --- | --- | --- | --- | --- | --- | --- |
| Did (s)he stop urinating? | 724 (35%) | 1,348 (64%) | 20  (1%) | 0 | 2,092 | |
| Did (s)he go to urinate more often than usual? | 1,061 (51%) | 1,015 (48%) | 15  (1%) | 1  (<1%) | 2,092 | |
| During the final illness did (s)he ever pass blood in the urine? | 404 (19%) | 1,622 (78%) | 65  (3%) | 1  (<1%) | 2,092 | |

**QUALITATIVE OBSERVATIONS**

- Cognitive interviewing demonstrated that question, *“Did (s)he have any urine problems?”* captured a variety of phenomena including:
  - Blood in the urine
  - Difficulty urinating
  - Discoloration of the urine
  - Incontinence
  - Pain while urinating
- Which symptoms respondents considered varied based on the age of the decedents. Specifically, respondents answering about young children commonly considered whether or not the child had difficulty urinating or urinated at all. For example, the notes of one Zambian mother describe:

*The respondent understood urine problems to mean a failure to pass urine. She said that the deceased [the respondent’s 6-month old daughter] did not have any urine problems, as she was able to pass urine before her death. “My daughter’s death was sudden—everything about her was ok.”*

- While all of the interpretations the cognitive interview respondents used appear, on their face, to be in-scope, there is a potential for response error if respondents limit their thinking to one of these, while excluding others. For example, one Zambian father answering about his child answered “No” and explained that he did not see blood in her urine. However, upon further probing he revealed that the decedent did not urinate in the days prior to her death, but that neither he nor his wife thought about this as a problem at the time.

## 10. ABDOMINAL PROBLEM

**ISSUE**

There is potential for redundancy and/or inconsistency across this series of questions. Consider the response patterns and the consistency of response to *“Did (s)he have any belly (abdominal) problem?”* and the subsequent required questions. Can this this question be eliminated or the series shortened in any way?

**Table 10.1 Question series, asked for children and adults**

| **Question** | **Comment** |
| --- | --- |
| Did (s)he have any belly (abdominal) problem? | Required |
| Did (s)he have belly (abdominal) pain? | Required |
| Was the belly (abdominal) pain severe? | If yes to first order question |
| For how long did (s)he have belly (abdominal) pain? | If yes to first order question |
| [Enter how long (s)he had belly (abdominal) pain in hours]: | If “hours” to second order question |
| [Enter how long (s)he had belly (abdominal) pain in days]: | If “days” to second order question |
| [Enter how long (s)he had belly (abdominal) pain in months]: | If “months” to second order question |
| Was the pain in the upper or lower belly (abdomen)? | If yes to first order question |
| Did (s)he have a more than usually protruding belly (abdomen)? | Required |
| For how long before death did (s)he have a more than usually protruding belly (abdomen)? | If yes to first order question |
| [Enter how long before death (s)he had a more than usually protruding belly (abdomen) in days]: | If “days” to second order question |
| [Enter how long before death (s)he had a more than usually protruding belly (abdomen) in months]: | If “months” to second order question |
| How rapidly did (s)he develop the protruding belly (abdomen)? | If yes to first order question |
| Did (s)he have any mass in the belly (abdomen)? | Required |
| For how long did (s)he have a mass in the belly (abdomen)? | If yes to first order question |
| [Enter how long (s)he had a mass in the belly (abdomen) in days]: | If “days” to second order question |
| [Enter how long (s)he had a mass in the belly (abdomen) in months]: | If “months” to second order question |

**QUANTITATIVE ANALYSIS**

Table 10.2 shows response patterns for “*Did (s)he have any belly (abdominal) problem?”* compared to the other required questions related to specific kinds of problems in this question series.

**Table 10.2 Response patterns for abdominal problem cross tabulated with subsequent abdominal questions**

|  | **Abdominal Pain** | | | | |
| --- | --- | --- | --- | --- | --- |
| **Abdominal**  **Problem** | **Yes**  **#**  **Row% Col%** | **No** | **Don’t Know** | **Refused** | **Total*** |
| **Yes** | 4,076  88%  87% | 459  10%  4% | 100  2%  18% | 0 | 4,635 (27%) |
| **No** | 613  5%  13% | 10,860  94%  96% | 140  1%  24% | 0 | 11,613 (70%) |
| **DK** | 15  4%  <1% | 53  13%  <1% | 333  83%  58% | 1 | 402 (2%) |
| **Refused** | 0 | 0 | 0 | 2 | 2 (<1%) |
| **Total** | 4,704 (28%) | 11,372 (68%) | 573 (3%) | 3 (<1%) | 16,652 |
|  | **Protruding Abdomen** | | | | |
| **Yes** | 1,466  31%  82% | 3,138  68%  21% | 33  1%  18% | 0 | 4,637 (28%) |
| **No** | 300  3%  17% | 11,260  97%  77% | 53  <1%  28% | 0 | 11,613 (70%) |
| **DK** | 26  7%  1% | 274  68%  2% | 101  25%  54% | 1 | 402 (2%) |
| **Refused** | 0 | 0 | 0 | 2 | 2 (<1%) |
| **Total** | 1,792 (11%) | 14,672 (88%) | 187 (1%) | 3 (<1%) | 16,654 |
|  | **Mass in Abdomen** | | | | |
| **Yes** | 810  17%  83% | 3,601  78%  24% | 225  5%  43% | 1 | 4,637 (28%) |
| **No** | 157  1.3%  16% | 11,292  97%  74% | 163  1.4%  32% | 1 | 11,613 (79%) |
| **DK** | 10  3%  1% | 262  65%  2% | 129  32%  25% | 1 | 402 (2%) |
| **Refused** | 0 | 0 | 0 | 2 | 2 (<1%) |
| **Total** | 977 (6%) | 15,155 (91%) | 517 (3%) | 5 (<1%) | 16,654 |

*Per the standard question skip pattern, all of these questions should be asked of the respondents, and it is expected that the total number would be the same for all questions; further work is needed to understand why there are discrepancies..

Table 10.3 demonstrates a cross tabulation of the abdominal problem responses with the three follow up first order questions about abdominal pain, protruding abdomen or mass in the abdomen

**Table 10.3 Crosstab of abdominal problem and any “Yes” response to subsequent abdominal questions**

|  | **Abdominal Pain, Protruding Abdomen, and**  **Mass in Abdomen** | | |
| --- | --- | --- | --- |
| **Abdominal**  **Problem** | **Yes to at least one**  **n (%)** | **No or Don’t know to all** | **Total** |
| **Yes** | 4,076 (88%) | 559 (12%) | 4,635 |
| **No** | 300 (3%) | 11,313 (97%) | 11,613 |
| **DK** | 27 (7%) | 375 (93%) | 402 |

**QUALITATIVE ANALYSIS**

- Respondents across both countries appeared to have consistent interpretations of “pain,” “protrusion,” and “mass,” respectively.
  - For pain, respondents most often considered whether or not the decedent cried (for children) or spoke about pain (for adult decedents).
  - Respondents understood “protrusion” as a swelling or enlargement of the belly or stomach.
    - For instance, one Moroccan wife who answered “Yes” to protruding abdomen explained that her husband’s stomach swelled about 6 months prior to his death, and that he was subsequently diagnosed with cancer.
  - Respondents understood “mass” to be an internal growth, such as a tumor.
    - For example, one Moroccan respondent who answered “Yes” to mass in the belly, noted that his father had multiple tumors in his intestine, which caused swelling and eventually external hardening before his death.
- Most respondents who answered “No” to abdominal problem went on to answer “No” to the other abdomen questions. Furthermore, all respondents who answered “Yes” to abdominal problem also answered “Yes” to at least one of the three subsequent abdominal questions.
  - Respondents considered a variety of phenomenon for “abdominal problem”, including
    - pain
    - swelling/protrusions
    - digestion (constipation and incontinence)
    - bleeding (stools, etc: cancer)
  - In cases when respondents answered “No” to abdominal problem but “Yes” to one of the three follow up question, respondents did not view the pain/protrusion/mass as a “Problem” associated with the death.
    - - a Zambian mother answering about her 4-month-old daughter answered “Yes” to 10200 (potruding belly): *Yes, the baby had a protruding belly in the last 3 days before death. [The respondent] said this was observed in a period when the child was failing to go to the toilet. She was asked do you think this kind of protrusion was due to sickness or because of failure to go to the toilet. She said this can’t be sickness because the baby had not been going to the toilet, so she thinks this is due to that problem of not going to the toilet. That’s why the belly was protruding.*
      - A Zambian father answering about his 5-month-old daughter who answered “Yes” to the belly pain question: *“She had usual digestion problems every baby has especially, during the first 3 months.” When asked how he knew that [the decedent’s] belly pains were the usual belly problems, he said that every baby experiences these pains in the first 3 months of their lives because they are still adjusting to the food that they are being fed. When asked if they had taken [his daughter] to the hospital, he said, “No I have 12 children, and they all experienced the same problem. Even if we took her to the clinic, we were just going to be told to breastfeed her frequently.”*

## 11. LUMPS

**ISSUE**

Does the ability to answer this question vary by respondent relationship to the deceased or whether or not the respondent lived with the deceased?

**Table 11.1 Question series, asked for children and adults**

| **Question** | **Comment** |
| --- | --- |
| Did (s)he have any lumps? | Required |
| Did (s)he have any lumps or lesions in the mouth? | If “yes” to first order question, for adults only |
| Did (s)he have any lumps on the neck? | If “yes” to first order question |
| Did (s)he have any lumps on the armpit? | If “yes” to first order question |
| Did (s)he have any lumps on the groin? | If “yes” to first order question |

**QUANTITATIVE ANALYSIS**

Tables 11.2 examines the response patterns of the first order question, “Di*d (s)he have any lumps?”* and the subsequent follow up questions.

**Table 11.2 Response patterns for question series on “lumps”**

| **Question** | **Yes** | **No** | **DK/Ref** | **Total** | |
| --- | --- | --- | --- | --- | --- |
| Did (s)he have any lumps? | 663 (4%) | 15,533 (94%) | 374 (2%) | 16,570 | |
|  |  | | | |  |
| Did (s)he have any lumps or lesions in the mouth? (adults only) | 209 (35%) | 383 (64%) | 4(1%) | 596 | |
| Did (s)he have any lumps on the neck? | 207 (31%) | 453 (68%) | 3 (1%) | 663 | |
| Did (s)he have any lumps on the armpit? | 105 (16%) | 547 (82%) | 11 (2%) | 663 | |
| Did (s)he have any lumps on the groin? | 167 (25%) | 465 (70%) | 31 (5%) | 663 | |

Table 11.3 shows the likelihood of responding “Don’t Know” to the lump question series by relationship of the respondent to the deceased and status of living with deceased. For the respondent relationship to the deceased, we defined “close” to include parent, child, sister or spouse.

**Table 11.3 Likelihood of responding “Don’t Know” to lump question series by family relationship and status of living with deceased**

| **Question** | **Close Family vs Other (ref)**  **Prevalence Ratio**  **(95% CI, p value)** | **Lived with Deceased vs**  **Other (ref)** |
| --- | --- | --- |
| Did (s)he have any lumps? | 0.51  (0.42-0.63, p<0.01) | 0.32  (0.26-0.41, p<0.01) |
| Did (s)he have any lumps or lesions in the mouth? | 0.74  (0.4 - 1.5, p=0.43) | 0.46  (0.05-4.4 p=0.43) |
| Did (s)he have any lumps on the neck? | 0.99  (0.98 - 1.2, p= 0.30) | 0.06  (0.01-0.61) p=0.03 |
| Did (s)he have any lumps on the armpit? | 0.3  (0.09 - 1.13, p=0.11) | 1.12  (0.14-8.65, p=1.0) |
| Did (s)he have any lumps on the groin? | 0.6  (0.3-1.2, p=0.18) | 0.58  (0.23-1.47 p=-.23) |

**QUALITATIVE OBSERVATIONS**

Cognitive interviews suggest that the question captured the correct construct; there was no confusion or response error that emerged.

- Respondents understood the term “lumps,” to be referring to outwardly visible growths under a decedent’s skin on their necks, arms, lymph nodes, etc.
- Most respondents explained their answer by noting that they did not notice any changes to the deceased’s body shape.
  - For instance, one Moroccan man answering about his sister in law explained that he did not observe any changes, and thus answered “No.” It appears that most of these respondents were thinking about lumps or visible masses on the neck, back, or armpits—areas that someone not intimately familiarly with the decedent’s body would be able to see.
- Other respondents included diagnoses from medical professionals in their responses
  - “The doctor did not diagnose any mass.”
- Results suggest that those living with the deceased may have more knowledge about lumps
  - ⅔ Don't know responses were from people living outside the household
  - All 5 “Yes” answers were from family living in the same household
  - Those living in the same household had more informed responses.
    - For instance, one Zambian father answering about his daughter spoke about a growth on the small of her back, which he determined did not count and answered “No.” The notes for this respondent explain:
      - *The respondents explained that his child did not have any lumps on her body and did not consider the growth she had as a lump. He said, “my daughter was born with a growth on her lower spinal cord, but she did not have any lumps on her body.*

## 12. VOMITING

**ISSUE**

Questions “*Did (s)he vomit?”* and *“To clarify: Did (s)he vomit in the week preceding the death?”* are very similar questions and both are asked of all respondents due to differing requirements by the automated algorithms. Can one question be eliminated? Also, clarification is needed on whether “*How long before death did s(h)e vomit?”* refers to the duration or timing of the vomiting, with possible clarification added to the question wording or as a hint with the question.

**Table 12.1 Question series, asked for neonates, children, and adults**

| **Question** | **Comment** |
| --- | --- |
| Did (s)he vomit? | Required |
| To clarify: Did (s)he vomit in the week preceding the death? | Required |
| How long before death did (s)he vomit? | If “yes” to first order question |
| [Enter how long before death(s)he vomited in days] | If “days” in second order question |
| [Enter how long before death(s)he vomited in months] | If “monts” in second order question |

**QUANTITATIVE ANALYSIS**

Table 12.3 shows a comparison of the response patterns for *“Did (s)he vomit?”* and *“To clarify: Did (s)he vomit in the week preceding death?”*

**Table 12.3 Crosstab of *“Did (s)he vomit?”* with *“To clarify: Did (s)he vomit in the week preceding death?”***

|  | **Did (s)he vomit in the week before death**  **#**  **Row%**  **Col%** | | | | |
| --- | --- | --- | --- | --- | --- |
| **Did (s)he vomit** | **Yes** | **No** | **DK** | **Ref** | **Total** |
| **Yes** | 3,127  73%  97% | 1,080  25%  14% | 72  2%  32% | 3  <1%  50% | 4,282 (38%) |
| **No** | 82  1%  3% | 6,700  98%  86% | 32  1%  14% | 0 | 6,814 (60%) |
| **DK** | 2  1%  <1% | 56  31%  <1% | 122  67%  54% | 1  1%  17% | 181 (2%) |
| **Ref** | 0 | 0 | 0 | 2  100%  33% | 2 (<1%) |
| **Total** | 3,211 (28%) | 7,836 (70%) | 226 (2%) | 6 (<1%) | 11,279 |

Table 12.4 shows the frequency distribution for responses to “*How long before death did (s)he vomit?”*.

**Table 12.4 Response frequency for “*How long before death did (s)he vomit?”* (n=4,282)**

| **0*** | **≤ 3 days** | **3-7 days** | **8-29 days** | **≥ 30** | **Unknown** |
| --- | --- | --- | --- | --- | --- |
| 320 (7%) | 1,818 (43%) | 800 (19%) | 324 (8%) | 991 (23%) | 29 (<1%) |

*Zero = less than 24 hrs

**QUALITATIVE OBSERVATIONS**

- In cognitive testing, overall, the questions captured slightly different constructs in terms of timing:
  - Most respondents understanding *“Did (s)he vomit?”* as asking about whether or not the decedent vomited in the immediate period before death—which varied from the hours before death to a few weeks;
  - Most understood “*To clarify: Did (s)he vomit in the week preceding death?”*  to be asking about the exact week before death. For example, one Zambian respondent who answered “No” to both questions explained that while her toddler son had vomited in his life, he did not do so in either the period he was sick (a few days) or the week before his death.
  - 20 respondents provided different answers to the two questions (n=8 No to 10188/Yes to 10189, n=4 No/Refuse, n=5 Yes/No, n=3 Yes/Refuse**)**
- “Yes” to *“Did (s)he vomit?”* but “No” to “*To clarify: Did (s)he vomit in the week preceding death?”*: Vomited with illness leading to death (cancer), but not in final week
  - Moroccan respondent who answered explained that for the first question, she was considering the whole time her husband was receiving treatment for his cancer and noted that the chemotherapy made him vomit. However, he had a very reduced diet in the last weeks of his life, and thus did not vomit in the week preceding his death.
  - Zambian respondent explained that while his brother vomited frequently during his illness, he did not do so in the final days of his life.
- “No” to *“Did (s)he vomit?”* but “Yes” to “*To clarify: Did (s)he vomit in the week preceding death?”*: Did not vomit in final stage of death (few days)
  - No reference period was given in the question text for *Did (s)he vomit?”* , and respondents largely understood it to be asking about the period of the final illness, there were other cases where “*To clarify: Did (s)he vomit in the week preceding death?”*: Did (with a set reference period of a week) represented the longer period of time than “*Did (s)he vomit?”*
  - One Moroccan respondent answering about her uncle noted that he stayed with her during his final few days of life, when he was very sick. And in that time the decedent did not vomit. However, the respondent went on to explain that her uncle did vomit in the last week of life—just not in the final three days that she was thinking about for the first question.

## 13. VIOLENCE

**ISSUE**

What is the consistency in responses to violence and self-inflicted injury for children?

There is a concern of under-reporting of suicide for children. It is a sensitive issue, but understanding the current response patterns may indicate if/how we can improve identification of child suicide.

**Table 13.1 Question series, asked for neonates (not stillbirths), children, and adults**

| **Question** | **Comment** |
| --- | --- |
| Did (s)he suffer from any injury or accident that led to her/his death? | Required |
|  |  |
| Was (s)he subject to violence (suicide, homicide, abuse)?  *Hint: (don’t say suicide for under-12-year olds)* | If “Yes/DK/Ref” to first order question and “No/DK/Ref” to road traffic accident |
| Was the injury accidental? | If “Yes/DK/Ref” to first order question |
| Was the injury self-inflicted? | If “No/DK/ref” to second order question, only asked for adult |
| Was the injury intentionally inflicted by someone else? | If “No/DK/Ref” to second order questions |

**QUANTITATIVE ANALYSIS**

**A.**

Table 13.2 shows the response patterns for this question series for deceased under 18 years old in the Primary dataset. Note that “*Was the injury self-inflicted?”* is not asked for children, so it is only available for 12-17 year-olds (=100).

**Table 13.2. Response patterns to violence question series in deceased under 18 years old (Primary dataset)**

| **Question** | **Yes** | **No** | **DK** | **Other** | **Total** |
| --- | --- | --- | --- | --- | --- |
| Did (s)he suffer from any injury or accident that led to her/his death? | 310  7% | 3,996  93% | 13  <1% | 1 refused  <1% | 4,320 |
| n=324 injury or accidents |  |  |  |  |  |
| Was (s)he subject to violence (suicide, homicide, abuse)? | 41  13% | 212  65% | 4  1% | 59 road traffic accidents (18%)  8 incorrect skips (3%) | 324 |
| Was the injury accidental? | 193  60% | 104  32% | 18  5% | 9 incorrect skips  3% | 324 |
| Was the injury self-inflicted?  Asked of adults only (age 12-17 yrs) | 18  18% | 43  43% | 7  7% | 32 accidental (32%) | 100 |
| Was the injury intentionally inflicted by someone else? | 24  7% | 88  27% | 18  6%% | 166 accidental (51%)  18 self inflicted (6%)  10 incorrect skips (3%) | 324 |

Figures A and B show consistency in reporting across indicators related to manner of death.

**
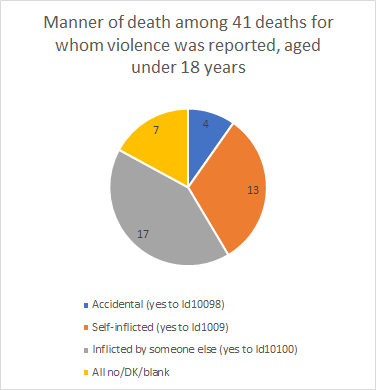

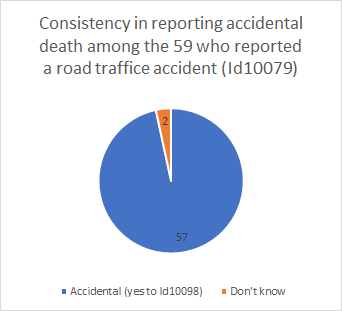
**

**Figure A Figure B**

**B.**

Using the reference dataset, where causes of death were independently assigned by physician certification of VA (PCVA), Table 13.3 looks at cause of death distributions for violent deaths among deceased ages 5-17 years old. Ages for those where self-inflicted injury was the assigned cause are provided in a footnote to the table.

**Table 13.3 Cause of death distribution for injury deaths among children 5-17 years, using independently assigned cause of death from South Africa data (n= 44)**

| **Injury Category** | **Number (%)** | **ICD Code (n): Cause** |
| --- | --- | --- |
| **Non-Violent/Accidental** | 21 (48%) | V03 (1): Pediatric Collision with a vehicle  V49 (1): passenger in transport accident  V099 (4): unspec transport accident  X09 (1): Exposure to uncontrolled fire  X33 (1): Victim of lightning  X59 (8): Exposure to unspec factor  V84 (1): Agricultural Vehicle Injury  W74 (2): Drowning/Submersion  W84 (1): Threat to breathing  W87 (2): Electric Current Exposure |
| **Self-Inflicted*** | 5 (11%) | X70 (4) : Hanging/strangulation  X64 (1): Intentional Self-poisoning |
| **Other violent (inflicted by someone else)** | 6 (12%) | Y09 (3): Unspec Assault  Y20: Assault by Strangulation  X99 (1): Assault by Sharp Object  X97 (1): Assault by Smoke or Fire |

* Ages of self inflicted : 11, 12, 14 (2), 17

**QUALITATIVE OBSERVATIONS**

- In cognitive testing, for “*Was (s)he subject to violence (suicide, homicide, abuse)?”* and “*Was it self-inflicted?”,* only two respondents answered “Yes” to the first question and went on to receive the second. One respondent who provided the potentially false positive response to the first question reported that he did not know whether or not the injury was self-inflicted, thinking still about the traffic incident. The respondent thinking about the violent death reported “No,” explaining that the decedent was apparently beaten to death by unknown people.
- Respondents across both countries appeared to understand the question to be asking about whether or not the decedent had been attacked or assaulted. No instances of potential suicide emerged across the sample, so the question of whether or not respondents equate suicide with “violence” remains unanswered.
- Most respondents in both Zambia and Morocco understood these questions as if they had no reference period—that is, they answered whether or not the decedent was ever subject to violence. This led to one potential false positive response, where a Moroccan respondent answered “yes” and explained that his relative had been in an aggressive traffic accident about four years prior to death. The decedent had not been subject to violence any time more recently. The only other respondent who answered “yes” to this question was thinking about violence as the proximate cause of death, reporting that the decedent was found with gouges across his face.
- Summary: “*Was (s)he subject to violence (suicide, homicide, abuse)?”* captures violence, but not necessarily within the time frame of death. There was no reported suicide.

## 14. SIZE AT BIRTH

**ISSUE**

For “*What was the weight (in grams) of the deceased at birth?”* and *“At birth, was the baby of usual size?”,* what is the consistency in responses to these questions? Do we need both questions?

*What was the weight (in grams) of the deceased at birth?”*. Categories of sizes do not mean the same across cultures. Consider using recorded weight and converting for use for COD assignment if available; only if the recorded weight is not available, use the categorical self-report of size.

**Table 14.1 Question series, asked for neonates and children under 1 year**

| **Question** | **Issue** |
| --- | --- |
| At birth, was the baby of usual size? | Required |
| At birth, was the baby smaller than usual, (weighing under 2.5 kg)? | If “No/DK/Ref” to first order question |
| At birth, was the baby very much smaller than usual, (weighing under 1 kg)? | If “Yes” to second order question |
| At birth, was the baby larger than usual, (weighing over 4.5 kg)? | If “No/DK/Ref” to first order question AND “no” to second order question |
| What was the weight (in grammes) of the deceased at birth? | Required |

**QUANTITATIVE ANALYSIS**

Table 14.3 provides the response frequencies for the baby size question series.

**Table 14.3 Response frequencies to baby size questions**

| **Question** | **Yes** | **No** | **DK** | **Total** |
| --- | --- | --- | --- | --- |
| At birth, was the baby of usual size? | 2,577 (69%) | 984 (26%) | 192 (5%) | 3,753 |
| At birth, was the baby smaller than usual, (weighing under 2.5 kg)? | 780 (61%) | 324 (25%) | 176 (14%) | 1,280 |
| At birth, was the baby very much smaller than usual, (weighing under 1 kg)? | 253 (28%) | 548 (62%) | 91 (10%) | 892 |
| At birth, was the baby larger than usual, (weighing over 4.5 kg)? | 37 (6%) | 384 (66%) | 159 (28%) | 580 |

Notes: 1) This analysis includes 335 cases where subsequent questions were answered that should have been skipped per the standard skip patterns.

Table 14.4 explores the plausibility of the reported weight in grams for “*What was the weight (in grams) of the deceased at birth?”* among those who answered “yes” to any of the baby size questions.

Table 14.4 Ranges of response for birth weight for those who answered “Yes” to baby size series questions

| **Question** | **What was the weight (in grammes) of the deceased at birth?** | | | | | | |
| --- | --- | --- | --- | --- | --- | --- | --- |
|  | **< 1000 grams *** | **1000 - 2499** | **2500 - 4500** | **>4500**** | **Implausible ***** | **Don’t Know** | **Total** |
| At birth, was the baby of usual size? | 28  (2%) | 181  (9%) | 991  (49%) | 29  (2%) | 87  (4%) | 683  (34%) | 1,999  (69%) |
| At birth, was the baby smaller than usual, (weighing under 2.5 kg)? | 69  (11%) | 263  (42%) | 22  (4%) | 0 | 36  (6%) | 236  (37%) | 626  (22%) |
| At birth, was the baby very much smaller than usual, (weighing under 1 kg)? | 66  (28%) | 36  (15%) | 4  (2%) | 1  (<1%) | 19  (8%) | 113  (47%) | 239  (8%) |
| At birth, was the baby larger than usual, (weighing over 4.5 kg)? | 1  (3%) | 1  (3%) | 10  (31%) | 7  (22%) | 2  (6%) | 11  (35%) | 32  (1%) |
| Total | 164 (6%) | 481 (17%) | 1,027  (35%) | 37 (1%) | 144 (5%) | 1,043  (36%) | 2,896 |

* Excludes those <100, which are considered “Implausible”

** Excludes those >6000, which are considered “Implausible”

*** Implausible: < 100 or > 6000; many of these weights are <100 gms suggesting these are kilogram recordings.

**QUALITATIVE OBSERVATIONS**

“*At birth, was the baby of usual size?”*

- Subjectivity of “usual weight”: As found in the previous cognitive evaluation of the verbal autopsy instrument,[1] asking about a subjective measurement of a baby at birth leads to a variety of interpretations. In the previous version of this question, tested in Nyanza Province, Kenya, the term “normal” was used, whereas the term “usual” was used in the version tested in Zambia and Morocco. While the term itself changed from the previous version of the questionnaire, the subjectivity remained:
  - No: “R (respondent) said no because D (deceased) was smaller compared to his other children at birth. He said D weighed 2kg and usual size is supposed to be at least about 3kg.”
  - Don’t know: ”I don’t know what the usual size of a baby is but a lot of children weigh between 3- 3.5kg, I have 12 children and one of them weighed 4.5kg and she still grew up without any complications.”
  - Yes: “I normally give birth at 11 months. Yes, because she had 3.6 kgs. what do you think is the normal size at birth, the respondent said according to me I give birth at 11 months with the other two daughters, though the deceased was delivered at 9 months with 3.6 kgs but her friends were born at 11 months the first at 4.3 kgs and the other child was 4.5 kilograms. So what is the usual normal size to you, she was further asked, she responded by saying above 3 point something I think is normal than two point something. The respondent did not give an upper limit of what her normal usual size is at birth.“
- Respondents assessed the “usual weight” through two main pathways: weight when born and duration of pregnancy.
  - Birth Weight: Many of these respondents judged the deceased child’s birth weight against some standard—typically in the 2kg to 4kg range.
    - Yes: “He said that the baby was of usual size, which he understood to be somewhere in the 2.5 to 3.5kg range. (this father reported that the birth weight was 3.2kg.)
    - Yes: and noted that his son was born one month premature, but was still 3.2kg at birth, which he said was a healthy weight.
  - Duration of pregnancy: Respondents who used this pattern focused more on when the baby was delivered than the actual birth weight of the baby. However, in most cases, respondents used the delivery date as a proxy indicator of the baby’s weight—in other words, a baby delivered full term should be a normal weight, whereas premature babies are underweight.
    - No: “For the “R”, his interpretation of ‘usual size’ was in terms of baby being born at 9 months of pregnancy but since his child was born before 9 months, he did not consider it as usual size.”
    - “Yes” She said the baby was of usual size because she was delivered at 7 months and her body was not that small, she looked bigger comparing with other children at the hospital the mother mentioned. When asked how much the baby weighted, she said 1kg and then when asked if that was the usual normal size to her comparing with the two children she has, she said yes

*“What was the weight (in grams) of the deceased at birth?”*

- 11/62 respondents did not know the birth weight. Reasons included:
  - Baby died at birth and wasn't weighted
  - Baby born at home and wasn't weighed
  - Never knew weight (“I’m not the mother”)
  - Doesn’t remember
- There were several ways in which respondents formed responses
  - Memory (most of respondents: “"R" did not have vaccination card but says that since child was born at the clinic she was weighed at birth and the wife told him the weight.”)
  - Estimates (“The newborn passed away a few hours after being born. The father confirmed that he took the newborn in his arms and that he weighed about 3000kg.s”
  - Card (2 respondents only: “when asked how he knew D's weight at birth R said he on D’s under five card.”

# LIMITATIONS

*Limitations and Considerations for Interpretation*

The following limitations should be taken into consideration when reviewing these findings:

With the exception of some CHAMPS data from Bangladesh, the available VA data only represent Sub-Saharan Africa at this time. The limited representation of the qualitative cognitive testing results (representing only Zambia and Morocco) versus the quantitative dataset is acknowledged.

While the most recent version of the WHO VA questionnaire (v1.5.2) contains a question on sex of the respondent, early versions were used for the datasets utilized in this analysis; accordingly, impact of the respondent sex on response is limited to the cognitive testing results, as this information was documented by cognitive testing.

There is some expected variation in the way the final VA instrument is applied in a given setting, due to different versions of the 2016 questionnaire being used, or other modifications that teams may make that have an impact on the electronic skip patterns. As such, some numbers do not track exactly as expected throughout this analysis. Analyses presented here are limited to those where such deviations had minimal to no impact, and notes are included where relevant to highlight deviations.

Percentages presented throughout the document are rounded and do not always equal 100%.
