# Supplementary material for: Mixed-methods analysis of select issues reported in the 2016 World Health Organization verbal autopsy questionnaire
Source: PLoS One. 2022 Oct 7;17(10):e0274304. doi: 10.1371/journal.pone.0274304 (PMC9543875; doi:10.1371/journal.pone.0274304)
Supplement: Attachment — Submitted filename: ResponsetoReviewers_12May2022.docx [file pone.0274304.s003.docx]

**Response to Reviewers**

Journal Requirements:

Please review your reference list to ensure that it is complete and correct. If you have cited papers that have been retracted, please include the rationale for doing so in the manuscript text, or remove these references and replace them with relevant current references. Any changes to the reference list should be mentioned in the rebuttal letter that accompanies your revised manuscript. If you need to cite a retracted article, indicate the article’s retracted status in the References list and also include a citation and full reference for the retraction notice.

Reviewers' comments:

6. Review Comments to the Author

Reviewer #1: Thank you for the responses and the methods and origin of the different data sources are now much clearer - again, really interesting work! The most minor of things - in the abstract, the final sentence in the background should be removed, as its a conclusion statement (although I think you added this on reviewer 3's request?), so maybe an editorial decision.

*Agree that as it was, it read like a conclusion; have revised some wording to explain how the results are framed in the paper and their purpose.*

Reviewer #2: The authors have done a careful job in responding to the 3 reviewers in-depth and relevant comments. This is an important manuscript which has hopefully already influenced work to continue to strengthen the VSA tool. However there remain a few targeted areas where the paper could be strengthened for reader understanding and impact

Abstract: The explanation of mixed methods is not needed, as now a commonly used and can be dropped to allow for more detail (like which 2 countries were used for the qualitative work)

*Have deleted explanation of mixed methods and restructured sentence in methods section to highlight the two countries where cognitive interviews were conducted.*

In the introduction the authors provide a nice description of the uses of mixed methods, but do not then state clearly which of these is being applied

*Have added details to highlight the approaches used in this study in lines 136-137. “The purpose of this investigation was to use a mixed methods approach triangulating cognitive interviewing and questionnaire performance findings to evaluate the performance of select questions in the 2016 version of the WHO questionnaire, as part of a broader questionnaire revision effort [1].”*

In the description of the GitHub data, they note "14 problematic issues for which solutions could be well-informed...."-I think these are 14 areas rather than grouping of issues? this should be clarified

*No change- we have reviewed all uses of the term “issues” in the paper, and we believe the term is correct in this case.*

While edited, it is still not clear to a new reader that only one of the datasets as physician validation. “The reference dataset included the VA data as in the primary dataset combined with cause of death information determined by physician review of the VA interview (PCVA or physician certified VA) from the South Africa National Cause of Death Validation Study [22].

This should be explicitly stated, as well as any differences in how the VA may have been administered in the reference set versus the other data.

*Have modified phrasing in lines 161-162 and 179-180 to clarify the addition of the PCVA information in the reference dataset; have also added a sentence in lines 181-183 to clarify that there was no difference in how the VA was administered.*

A reference on cognitive interviewing is needed. *Reference added in line 200, at beginning of qualitative data section.*  In addition, were the respondents interviewed also being interviewed for the VA? Were any changes made or requested after the cognitive interviewing when issues were identified in interpretation? *Added clarification in lines 210-211.* As noted by a reviewer-a COREQ checklist should be completed as an appendix. Given the importance of the qualitative and emphasis on mixed methods, there remains a lack of description of the qualitative analysis (as was identified by a previous reviewer). This should be corrected and a reference for the methodology (and why chosen) added

*The analysis presented in this paper (starting in line 246) is of secondary data—triangulating findings from various data sources. While a brief description of the analysis of the primary qualitative data is included in the data collection section (lines 232-238), that is not the focus of this paper, and the organization and content is targeted accordingly.*

In results, were there any differences in the 4 main areas based on either age group of the deaths (ex. Neonate versus older adults) and across countries? Any identified issues for example with linguistic or cognitive translation of questions?

*We unfortunately did not have sufficient data to explore these differences; have added a statement on this limitation at the end of the limitations section.*

How was the reference dataset used beyond the use in injury?

*It was used in the analysis of three of the fourteen issues; this detail as been added in lines 183-184. “The reference dataset contributed to analysis for three issues, including the medical diagnosis questions, injury, and violence.”*

The discussion is much stronger after revisions. I still did not see any discussion about the reference dataset (was that for accuracy of the VA versus physician dx and if so, results and discussions?). I would only add into limitations that differences based on age group of the deceased should be included.

*The reviewer is correct that the reference dataset was used to look at the accuracy of the physician dx questions and response consistency with the associated symptom questions; this item was not one of the select issues that we gave more detail about, but detail on this use has now been added in lines 181-184 as noted above. Have added the requested statement in the limitations about differences based on age group.*

Reviewer #4: Nichols et al. conducted a mixed-method analysis titled, “Mixed-Methods Analysis of Selected Issues Reported in the 2016 World Health Organization Verbal Autopsy Questionnaire”, in which they show that WHO VA questionnaire requires revisions and clarifications to improve the respondents understanding of the questionnaire. In my opinion, the study can be improved by incorporating the following points:

1. The authors have not mentioned regarding how they computed the cross-tabulations data and evaluated the significance of their results, such as with a chi square test. Also mention the p value that was considered significant.

*Details added in lines 254-255. “The impact of respondent characteristics on ability to provide substantive responses was evaluated by calculating prevalence ratios, 95% confidence intervals and p-values using the chi-squared test with p<0.05 considered significant.”*

2. As the data was collected by the field team, who were included in the field teams, such as doctors or nurses? More details can be mentioned.

*Have added details in lines 166-167.* “*Project teams that had completed at least 1,000 VAs conducted by field interviewers (typically, though not always, a community health employee) using the 2016 WHO VA questionnaire and who agreed to contribute their data for the exercise submitted data to the analysis team.”*

3. In the discussion (Line 411 – 413) when the authors have compared injury related symptom question in death due to injury vs non-injury, they should elaborate more on the reason why response was lower.

*Have added clarify in lines 424-425: “These findings suggest that deaths with indication of injury most often have no other symptoms to report and eliminating the rest of the questionnaire or using an abbreviated set of symptom questions to screen for non-injury or maternal causes after report of an injury could be applied.”*

4. In WHO VA version 1.5.2 and 1.5.3, which particular respondent characteristics affect the reliability of the response? This can be mentioned to improve this part of the discussion as the authors have already highlighted that this is a sensitive issue.

*Lines 448-451 describe the two respondent characteristics that were studied in this work; have added emphasis on this in line 451. “In our investigation, gendered questions, such as the female reproductive health questions, yielded more substantive, “Yes” or “No”, responses when provided by respondents who were either close family members or lived with the deceased—the two respondent characteristics that were evaluated in this work.”*

5. Please add a reference for WHO VA 1.5.2/3.

*Added.*

6. The results state that clarity of construct was the ability to understand the terminologies and the intention of the question. However, the discussion lacks any explanations regarding the findings of clarity of construct and how this affects the questionnaire and the responses by the participants.

*Noted- have added text in lines 436-439 to cover this topic as the others have been covered. “Likewise, questions with similar but distinct terminology can results in incorrect association of intended constructs, as was observed among the medical diagnosis questions. Clarity of the construct of interest can be supported by strategic organization of questions to provide a questionnaire flow that aids the respondent in tracking the distinct constructs of interest.”*

7. It is mentioned that an open narrative regarding the circumstances of death is also collected but it can be mentioned that what are the benefits of this? Such as can this type of questioning give more detailed and qualitative data? The authors may give more references here to support this claim.

*Have added detail and a reference on this point in lines 442-445. “ Open narratives have been reported as a way to build rapport with respondents, improving the ability to collect quality information [29].”*

8. The manuscript needs to be proofread for grammatical mistakes

*A full review has been completed.*

9. The conclusion of the study needs to be improved. The authors may include particular summarized findings from the study as a properly written conclusion affects the readers’ interest.

*Have reframed the concluding statements.*

END OF REVIEW
